# Galectin-3 Deletion Reduces LPS and Acute Colitis-Induced Pro-Inflammatory Microglial Activation in the Ventral Mesencephalon

**DOI:** 10.3389/fphar.2021.706439

**Published:** 2021-08-18

**Authors:** Ana M. Espinosa-Oliva, Pablo García-Miranda, Isabel María Alonso-Bellido, Ana E. Carvajal, Melania González-Rodríguez, Alejandro Carrillo-Jiménez, Arturo J. Temblador, Manuel Felices-Navarro, Irene García-Domínguez, María Angustias Roca-Ceballos, María D. Vázquez-Carretero, Juan García-Revilla, Marti Santiago, María J. Peral, José Luis Venero, Rocío M. de Pablos

**Affiliations:** ^1^ Instituto de Biomedicina de Sevilla (IBiS), Hospital Universitario Virgen Del Rocío/CSIC/Universidad de Sevilla, Departamento de Bioquímica y Biología Molecular, Facultad de Farmacia, Universidad de, Sevilla, Spain; ^2^ Departamento de Fisiología, Facultad de Farmacia, Universidad de Sevilla, Sevilla, Spain; ^3^ Neuroplasticity and Neurodegeneration Laboratory, Ciudad Real Medical School, CRIB, Universidad de Castilla-La Mancha, Ciudad Real, Spain; ^4^ Laboratory of Virology and Chemotherapy, Department of Microbiology, Immunology and Transplantation, Rega Institute for Medical Research, Leuven, Belgium

**Keywords:** galectin-3, microglia, Parkinson’s disease, peripheral inflammation, neuroinflammation

## Abstract

Parkinson’s disease is a highly prevalent neurological disorder for which there is currently no cure. Therefore, the knowledge of risk factors as well as the development of new putative molecular targets is mandatory. In this sense, peripheral inflammation, especially the originated in the colon, is emerging as a predisposing factor for suffering this disease. We have largely studied the pleiotropic roles of galectin-3 in driving microglia-associated immune responses. However, studies aimed at elucidating the role of galectin-3 in peripheral inflammation in terms of microglia polarization are lacking. To achieve this, we have evaluated the effect of galectin-3 deletion in two different models of acute peripheral inflammation: intraperitoneal injection of lipopolysaccharide or gut inflammation induced by oral administration of dextran sodium sulfate. We found that under peripheral inflammation the number of microglial cells and the expression levels of pro-inflammatory mediators take place specifically in the dopaminergic system, thus supporting causative links between Parkinson’s disease and peripheral inflammation. Absence of galectin-3 highly reduced neuroinflammation in both models, suggesting an important central regulatory role of galectin-3 in driving microglial activation provoked by the peripheral inflammation. Thus, modulation of galectin-3 function emerges as a promising strategy to minimize undesired microglia polarization states.

## Introduction

Parkinson’s disease (PD) is the second most prevalent neurodegenerative disorder with 1 million people affected all over the word (de Rijk et al., 1997). This neurological disorder results from the loss of dopaminergic neurons of the substantia nigra pars compacta (SNpc), which leads to the depletion of dopamine levels in striatum (Kordower et al., 2013). Striatal dopamine loss is responsible for the cardinal symptoms of PD, including tremor, bradikinesia, postural instability and rigidity along with a variety of non-motor symptoms (Kumaresan and Khan 2021). In an effort to decipher the underlying causes of this disease, the scientific community has shown that mitochondrial dysfunction, oxidative stress, excitotoxicity and alterations in the ubiquitin-proteasome system that leads to α-synuclein aggregation, cooperate in the dopaminergic neuronal death that takes place in PD (Gundersen 2020). However, to date there is no treatment capable of curing, or at least, slowing down the progress of this disease. For this reason, it is imperative to know the risk factors of PD as well as discover new molecular targets that improve the treatment options.

Reactive microgliosis, a process long considered secondary to neurodegeneration, is a characteristic typically associated with neurodegenerative diseases, including PD (Heneka et al., 2015). However, with the development of genome-wide association studies (GWAS), convincing evidence has been found on the probable direct involvement of microglia in neurodegeneration processes. Thus, different GWAS studies have identified several risk genes associated with PD strongly related to the innate immune system, including the triggering receptor expressed on myeloid cells 2 (TREM2) (Nalls et al., 2014); (Rayaprolu et al., 2013); (Hirsch and Hunot 2009). Moreover, recent massive transcriptomic studies have provided fundamental clues about the molecular profile of microglia, the immune cells of the central nervous system (CNS), under homeostatic conditions (Butovsky et al., 2014), aging (Hickman et al., 2013); (Galatro et al., 2017); (Grabert et al., 2016) and various models of neurodegeneration (Keren-Shaul et al., 2017); (Krasemann et al., 2017); (Mathys et al., 2017). Recently, Friedman and colleagues have compared the genome-wide transcriptional responses of brain myeloid cells obtained from diverse neuropathological models and identified modules of genes that show similar responses in multiple settings. Among these modules authors highlighted the proliferative, interferon-related, core neuro-degeneration-related and LPS-related modules (Friedman et al., 2018). All these transcriptomic studies surprisingly found a very similar transcriptional pattern in neurodegenerative conditions, characterized by the selective induction of multiple genes, including Itgax (Cd11c), Axl, Clec7a, MHC class II and Lgals3 (galectin-3; Gal3). Gal3 is the only known member of the chimera-type family of galectins. It is involved in the inflammatory response, and its expression is increased in microglial cells upon various neuroinflammatory stimuli (Lalancette-Hébert et al., 2012); (Satoh et al., 2011; Wesley et al., 2013).

We have recently demonstrated that Gal3 can bind to and activate different microglial receptors including TREM2 (Boza-Serrano et al., 2019) and Toll-like receptor (TLR)-4 (Burguillos et al., 2015). We have also shown that Gal3 plays a role in α-synuclein-induced microglial activation (Burguillos et al., 2015); (Boza-Serrano et al., 2014) and that Gal3 regulates inflammatory response in Alzheimer’s disease (Boza-Serrano et al., 2019). These data point out Gal3 as a candidate of enormous relevance in the process of microglial activation associated with neurodegeneration and, therefore, its possible involvement in the neuroinflammation that takes place in PD deserves special attention.

Regarding the risk factors of PD, it has been proposed that peripheral inflammation could induce an inflammatory environment in the brain that eventually could lead to neurodegeneration (Herrera et al., 2015). Thus, peripheral injections of the inflammogen lipopolysaccharide (LPS) increase pro-inflammatory cytokine production and induce the death of dopaminergic terminals in the striatum (Beier et al., 2017). Moreover, our group and others have pointed out the possibility that different forms of peripheral inflammation, especially that induced by gut inflammation, increase the death of nigral dopaminergic neurons (Hernández-Romero et al., 2012); (Villarán et al., 2010); (Garrido-Gil et al., 2018); (Herrera et al., 2015). Intriguingly, recent cohort studies have shown an association between PD and inflammatory bowel diseases (IBDs), one of whose typical feature is long-lasting systemic inflammation (Villumsen et al., 2019); (Wan, Zhao, and Wu 2020); (Weimers et al., 2019); (Zhu et al., 2019); (Brudek 2019). Actually, the gut-brain axis hypothesis of PD is gaining a lot of attention, as supported by the exponential increase in the number of papers referring to this topic in the last 10 years (for a review, see (Kaushal, Singh, and Singh 2021).

With all these precedents, the aim of this study was to investigate whether two different models of acute peripheral inflammation are able to induce neuroinflammation in the ventral mesencephalon and the ability of Gal3 to regulate brain inflammation in response to both systemic LPS and oral dextran sulphate sodium (DSS) model of ulcerative colitis (UC).

## Material and Methods

### Animals and Treatments

12–16 week-old wild type (WT) and Gal3 null mutant mice (Gal3KO) on the C57BL/6 background (Colnot et al., 1998) were obtained from Dr. T. Deierborg from Lund University. Both colonies were maintained at the Centre of Production and Animal Experimentation of the University of Seville. Animals were housed at constant room temperature (RT) of 22 ± 1°C and relative humidity (60%), with a 12 h light-dark cycle and *ad libitum* access to food and water. Experiments were carried out in accordance with the Guidelines of the European Union Directive (2010/63/EU) and Spanish regulations (BOE 34/11370–421, 2013) for the use of laboratory animals; the study was approved by the Scientific Committee of the University of Seville.

For the LPS treatment, 40 mice were divided into four groups: 1) WT group, control WT animals injected intraperitoneally (i.p.) with 100 µl 0.9% sterile saline; 2) WTLPS group, WT animals injected i.p. with 100 µL LPS (Sigma-Aldrich, St. Louis, MO, United States) in 0.9% sterile saline, at a dose of 1 μg/g for four consecutive days; 3) Gal3KO group, Gal3KO animals injected i.p. with 100 µL 0.9% sterile saline; and 4) Gal3KOLPS group, Gal3KO animals injected i.p. with 100 µL LPS in 0.9% sterile saline, at a dose of 1 μg/g for four consecutive days. Animals were sacrificed 5 days after the first LPS injection.

The acute colitis was induced by oral administration of DSS (molecular weight 36,000–50,000 Da; MP Biomedicals, LLC) at a concentration of 4% in tap water. A total of 40 mice organized into four groups were used: 1) WT group, control WT animals drinking tap water; 2) WTDSS group, WT animals drinking DSS for 10 days; 3) Gal3KO group, Gal3KO animals drinking tap water; and 4) Gal3KODSS group, Gal3KO animals drinking DSS for 10 days. Animals were sacrificed 10 days after the beginning of the treatment.

In order to carry out a clinical evaluation of the inflammation generated by the DSS, the animals were monitored, at the beginning and end of the treatment, based on the named Disease Activity Index (DAI). DAI was assessed according to a standard scoring system, and body weight, stool consistency and rectal bleeding were recorded (Cooper et al., 1993). Loss in body weight was scored as: 0, no weight loss; 1, weight loss of 1–5% from baseline; 2, 5–10%; 3, 10–20%; and 4, >20%. For stool consistency, a score of 0 was assigned for normally formed pellets, 1 for soft pellets not adhering to the anus, two for very soft pellets adhering to the anus and three for liquid stools adhering to the anus. For bleeding, a score of 0 was assigned for no blood, 1 for small spots of blood in stool and dry anus, two for large spots of blood in stool and through anal orifice and three for gross bleeding and largely around the anus. These scores are added together and divided by three, resulting in DAIs ranging from 0 (healthy) to 3 (maximal disease activity). Body weight loss was calculated as the percentage of the initial weight (day 0).

The colon inflammation was assessed by colon length shortening, histological score and by the mRNA expression levels of the pro-inflammatory cytokines interleukin (IL)-1β and tumor necrosis factor (TNF)-α, as described (Carvajal, et al., 2017b). Following sacrifice, the colons were removed, washed with ice-cold saline solution, and measured. For histological analysis, the distal colons were fixed by overnight incubation with phosphate buffer saline (PBS) (in mM, 137 NaCl, 2.7 KCl, 10 Na2HPO4 and 1.8 KH2PO4 pH 7.4) containing 4% para-formaldehyde. Tissues were embedded in paraffin and cut into 5 µm-thick sections that were stained with hematoxylin/eosin. The analysis was performed in a blinded fashion by a validated method (Cooper et al., 1993). Colon damage was graded based on destruction of epithelium, dilatation of crypts, loss of goblet cells, inflammatory cell infiltrate, oedema and crypt abscesses. Each of these parameters is scored on a scale of 0 to 3 and added together to obtain a total severity scoring.

### Cell Proliferation Rate

Epithelial cell proliferation rate was quantified by measuring the incorporation into DNA of 5-bromo deoxyuridine (BrdU) as previously described (Carvajal, et al., 2017a). Briefly, mice received an i.p. injection of BrdU (120 mg/kg body weight) and were sacrificed 90 min later. BrdU was detected by immunohistochemistry using an anti-BrdU antibody (1:300; Sigma-Aldrich). Antibody binding was visualized with a biotinylated antibody followed by immunoperoxidase staining. Vectastain ABC peroxidase kit (Vector) and 3,3′-diaminobenzidine were used. The slides were rinsed, mounted and photographed with a Zeiss Axioskop 40 microscope equipped with a SPOT Insight V 3.5 digital camera. Acquired images were analyzed by using Spot Advance 3.5.4.1 program (Diagnostic Instrument, Inc.,). The number of labelled cells was determined in at least 15 crypts well oriented longitudinally per mouse. Crypt cell proliferation rate is expressed as the number of BrdU-positive cells per crypt.

### Goblet Cells Staining

The identification and quantification of mature goblet cells in the epithelium of the distal colon was performed by immunohistochemical detection using an anti-mucin-2 (MUC-2) antibody (1:25; Santa Cruz Biotechnology), as described above. The number of positive cells was determined in at least 15 crypts longitudinally well oriented per mouse and the results are expressed as the number of MUC-2 positive cells per crypt.

### Real-Time Quantitative Reverse Transcription PCR

SN, striatum and distal colon were dissected from each mouse after 4 (for LPS) or 10 (for DSS) days of treatment, snap frozen in liquid nitrogen and stored at −80°C. Total RNA was extracted using RNeasy^®^ kit (Qiagen). cDNA was synthesized from 1 μg of total RNA using QuantiTect^®^ reverse transcription kit (Qiagen) in 20 μL reaction volume as described by the manufacturer. RT-qPCR was performed with iQ™SYBR^®^ Green Supermix (Bio-Rad Laboratories), 0.4 μM primers and 1 μL cDNA. Controls were carried out without cDNA. Amplification was run in a Mastercycler^®^ ep realplex (Eppendorf) thermal cycler at 94°C for 3 min followed by 35 cycles of 94°C for 30 s, 55°C for 45 s, and 72°C for 45 s, followed by a final elongation step at 72°C for 7 min. Following amplification, a melting curve analysis was performed by heating the reactions from 65 to 95°C in 1°C intervals while monitoring fluorescence. Analysis confirmed a single PCR product with the melting temperature. β-actin served as a reference gene and was used for sample normalization. The primer sequences for Gal3, TNF-α, IL-1β, IL-6, CXCL10, NOS2, COX2, IL-10 and β-actin are shown in Table 1. The cycle at which each sample crossed a fluorescence threshold, Ct, was determined, and the triplicate values for each cDNA were averaged. Analyses of RT-qPCR were performed using a comparative Ct method integrated in a Bio-Rad System Software.

**TABLE 1:** Sense and antisense sequences of the primers used for the analysis of mRNA expression by qPCR. Abbreviations: Gal3, galectin-3; TNF-α, tumor necrosis factor *α*; NOS2, nitric oxide synthase two; IL, interleukin; CXCL10, C-X-C motif chemokine 10; COX2, cyclooxygenase-2.

Gene	Forward (5′- 3′)	Reverse (5′- 3′)
β-actin	5′-CCA​CAC​CCG​CCA​CCA​GTT​CG-3′	5′-CCC​ATT​CCC​ACC​ATC​ACA​CC-3′
Gal3	5′-GAT​CAC​AAT​CAT​GGG​CAC​AG-3′	5′-GTG​GAA​GGC​AAC​ATC​ATT​CC-3′
TNF-α	5′-TGC​CTA​TGT​CTC​AGC​CTC​TTC-3′	5′-GAG​GCC​ATT​TGG​GAA​CTT​CT-3′
NOS2	5′-CTT​TGC​CAC​GGA​CGA​GAC-3′	5′-TCA​TTG​TAC​TCT​GAG​GGC​TGA​C-3′
IL-6	5'-GAC​AAA​GCC​AGA​GTC​CTT​CAG​A-3′	5'-AGG​AGA​GCA​ATT​GGA​AAT​TGG​GG-3′
IL-1β	5′-TGT​AAT​GAA​AGA​CGG​CAC​ACC-3′	5′-TCT​TCT​TTG​GGT​ATT​GCT​TGG-3′
IL 10	5′-CCA​AGC​CTT​ATC​GGA​AAT​GA-3′	5′-TTT​CAC​AGG​GAG​AAA​TCG-3′
CXCL10	5′-AAG​CAT​GTG​GAG​GTG​CGA​C-3′	5′-CTA​GGG​AGG​ACA​AGG​AGG​GT-3′
COX2	5′-CCA​GGC​CGA​CTA​AAT​CAA​GC-3′	5′-GGA​CAA​TGG​GCA​TAA​AGC​TAT​GG-3′

### Immunohistological Evaluation

For Iba-1 and CD68 staining, animals were perfused through the heart under deep anesthesia (isoflurane) with 150–200 ml of 0.9% sterile saline. After perfusion, brains were removed and fixed in 4% para-formaldehyde in phosphate buffer, pH 7.4. Then they were cryoprotected serially in sucrose in PBS, pH 7.4, first in 10% sucrose for 24 h and then in 30% sucrose until sunk (2–5 days). The brains were then frozen in isopentane at −80°C. Incubations and washes were in Tris-buffered saline (TBS) or PBS, pH 7.4. All work was performed at RT. Coronal sections (20 µm) were cut on a cryostat at −20°C and mounted in gelatin-coated slides. Sections were washed and then treated with 0.3% hydrogen peroxide in methanol for 20 min and washed again. Sections were then incubated in a solution containing TBS and 1% goat serum (Vector) for 60 min in a humid chamber. Slides were then drained and further incubated with rabbit-derived anti-Iba-1(1:500, Wako) and mouse-derived anti-CD68 (1:100, Invitrogen) in TBS containing 1% serum and 0.25% Triton-X-100 for 24 h. The immunostaining continued incubating the sections for 2 h with biotinylated goat anti-rabbit IgG (1:200, Vector), for Iba-1 or with goat anti-mouse IgG (1:200, Vector) for CD68. The secondary antibody was diluted in TBS containing 0.25% Triton-X-100 and its addition was preceded by three 10 min rinses. Sections were then incubated with the VECTASTAIN®-Peroxidase solution (1:100; Vector). The peroxidase was visualized with a standard diaminobenzidine/hydrogen reaction for 5 min.

### Immunohistochemistry Data Analysis

For the measurement of Iba-1 and CD68 immunoreactivity, we used the AnalySIS imaging software (Soft Imaging System GmbH) coupled to a Polaroid DMC camera (Polaroid) attached to a light microscope (Leica Mikroskopie). Cells showing Iba-1 or CD68 immunoreactivity were counted by using five sections per animal systematically distributed through the brain anterior–posterior axis. In each section, a systematic sampling of the area occupied by the Iba-1/CD68 positive cells was made from a random starting point with a grid adjusted to count five fields per section. An unbiased counting frame of known area (40 μm × 25 µm = 1,000 μm^2^) was superimposed on the tissue section image under a ×40 objective. The different types of Iba-1-positive cells (displaying different shapes depending on their activation state) in the different treatment conditions were counted as a whole. The entire z-dimension of each section (20 µm in all animals) was sampled; no guard zone was used; hence, the section thickness sampling fraction was 1.

### Immunofluorescence

Animals were perfused and sections were prepared as described above. Incubations and washes for all the antibodies were in PBS, pH 7.4. All work was done at RT, unless otherwise noted. Sections were blocked with PBS containing 5% BSA and 1% Triton X-100 for 2 h. The slides were then incubated overnight at 4°C with the primary antibody: sheep-derived anti-Tyrosine hydroxylase (anti-TH, 1:1,000, Novus), rabbit-derived anti-caspase 3 (1:250, Cell Signaling; 1:250), rabbit-derived anti-Iba1 (Wako; 1:1,000) and goat-derived anti-galectin-3 (R&D Systems; 1:500). Primary antibodies were diluted in PBS containing 1% BSA and 1% Triton X-100. After three washes in PBS, sections were incubated with the secondary antibodies, Alexa Fluor^®^ 488, Alexa Fluor^®^ 546 and Alexa Fluor^®^ 647 (Invitrogen; 1:500), for 2 h at room temperature in the dark. Fluorescence images were acquired using a confocal laser scanning microscope (Zeiss LSM seven DUO) and processed using the associated software package (ZEN 2010). Iba-1/galectin-3 and TH/Caspase three co-localizing cells were visualized with Image-J software.

### Fluoro Jade C

Before use, slides were dried at RT for 10 min, and washed in 80% ethanol for 5 min and then in 70% ethanol for 2 min. Then slides were washed for 2 min in distilled water and incubated in a solution of 0.06% potassium permanganate solution for 10 min, on a shaker table to ensure consistent background suppression between sections. The slides were then washed in distilled water for 2 min. The staining solution was prepared as described by the manufacturer using a 0.0001% solution of Fluoro jade C (Histo-Chem Inc., Jefferson AR). Hoechst was added to the working solution in a concentration of 1:1,000. After 10 min in the staining solution, the slides were rinsed for 1 min in each of three distilled water washes. Excess water was removed by briefly draining the slides vertically on a paper towel. The slides were then placed on a slide warmer at 50°C for 30 min in darkness (until they were fully dry). The dried slides were cleared by immersion in xylene for 1 min before coverslipping with DPX (VWR International Inc., Poole, England), a non-aqueous, non-fluorescent plastic mounting media.

### Statistical Analysis

Results are expressed as mean ± SD. Means were compared by one-way ANOVA followed by the Fisher’s LSD test for post hoc multiple range comparisons. We also performed a multifactor ANOVA to analyze the interactions between the two factors included in the comparisons made in the study: genotype (WT and KO animals) and treatment (control and DSS). These results are summarized in Supplementary Appendix S1, S2. An alpha level of 0.05 was used. The Statgraphics Plus 3.0 statistical package was used for the analyses.

## Results

### Dextran Sulphate Sodium Treatment Induces Acute Colitis

**FIGURE 1:**
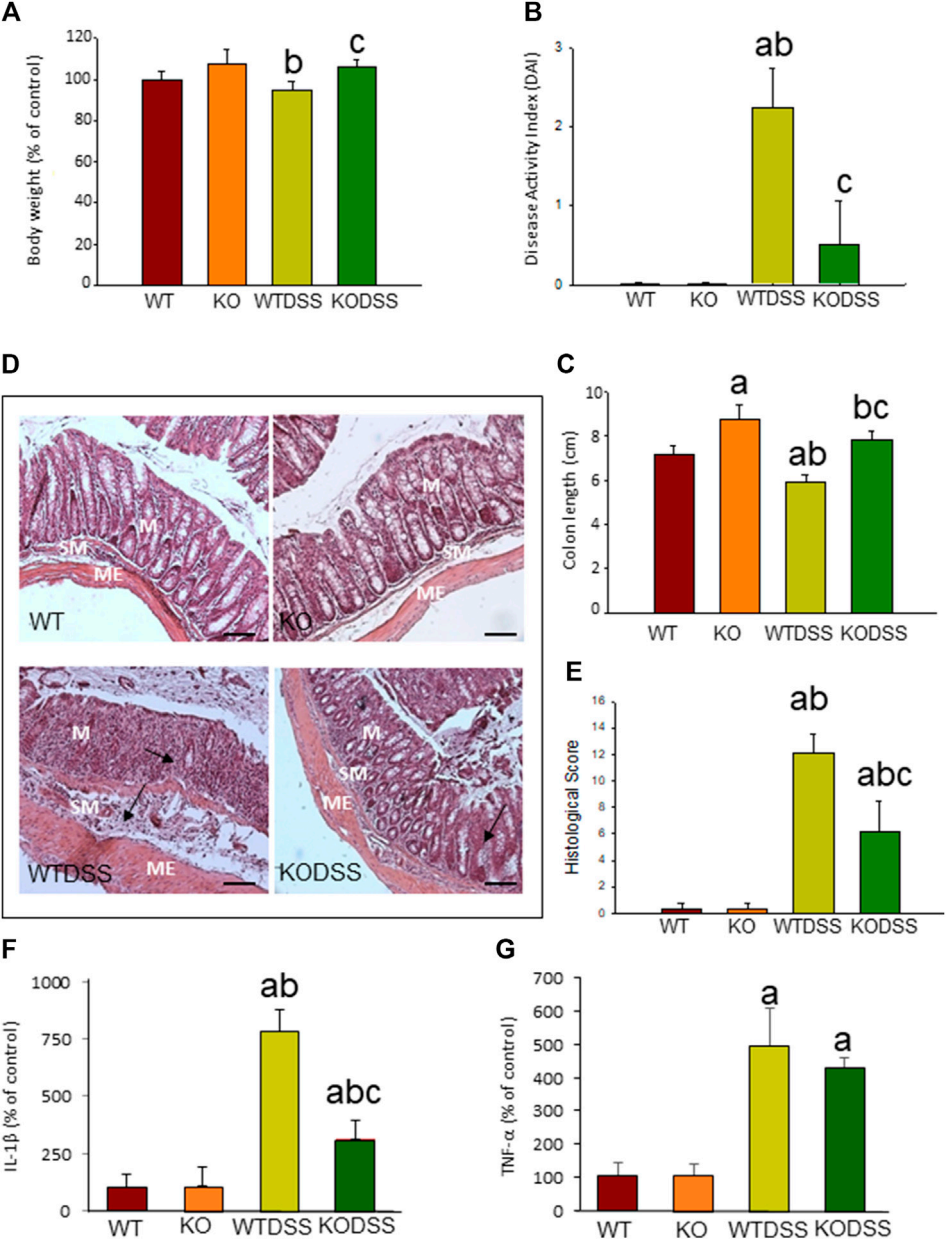
Evaluation of the DSS-induced inflammation in WT and Gal3KO mice. The mice were subjected to 4 % DSS in the drinking water for up to 10 days. Clinical symptoms and indicators of colonic inflammation were assessed at the end of the treatment. Body weight expressed as percentage of control (WT mice) **(A)**; disease activity index (DAI) **(B)**; Length of colon **(C)**; Representative photographs of histological analysis **(D)** performed with hematoxylin/eosin staining in distal colon sections. Scale bar: 200 µm. Alterations such as loss of structure of colonic crypts and infiltration of immune cells are indicated by arrows (M: mucosa; SM: submucosa; ME: muscularis externa); Histological score **(E)**; mRNA relative expression of IL-1β **(F)** and TNF-*α* **(G)** in distal colon expressed as percentage of control (WT mice). Results are mean ± SD of 10 mice per experimental group. Statistical analysis: One-way ANOVA followed by the Fisher’s LSD post hoc test for multiple comparisons was used, with *α* = 0.05: **(a)**, compared with the WT group; **(b)**, compared with the KO group; **(c)**, compared with the WTDSS group; *p* < 0.05 for body weight and mRNA expression, *p* < 0.001 for colon length, DAI and histological score. Abbreviations: WT, wild type mice; WTDSS, wild type mice treated with DSS; KO, Gal3 knockout mice; KODSS, Gal3 knockout mice treated with DSS.

Clinical evaluation of the animals and analysis of the colon damage at the end of the treatment were made, as described in Methods. All DSS-treated mice developed acute colitis with clinical symptoms and colon damage. Figure 1A shows that, although the body weight of WTDSS animals did not decrease compared to WT mice, there were differences in the DAI score used for clinical evaluation. DAI score revealed that, while control animals (WT) had 0 points, WTDSS mice were affected, reaching a punctuation of 2.25 ± 0.5 (*p* <0.001; Figure 1B; see Supplementary Appendix S1 for multifactor ANOVA analysis). We next evaluated the colon length shortening and histological scoring as indicators of colonic inflammation. Figure 1C reveals that the colon length significantly decreased in WTDSS mice compared with WT animals (*p* <0.001; see Supplementary Appendix S1 for multifactor ANOVA analysis). Histological analysis also showed colon inflammation in WTDSS mice (Figures 1D,E). We also carried out RT-qPCR experiments to analyze mRNA expression levels of the pro-inflammatory cytokines IL-1β and TNF-α in the distal colon. Figures 1F,G show an increase in both cytokines with the DSS treatment in WT animals (*p* <0.05, compared to the control WT animals; see Supplementary Appendix S1 for multifactor ANOVA analysis).

### The Absence of *Gal3* Prevents the Development of Severe Dextran Sulphate Sodium -Induced Acute Colitis

To elucidate whether the absence of *Gal3* modifies the disease severity in acute colitis, we compared the response between WT and Gal3KO mice after 10 days of DSS treatment. Figure 1A shows that there is a statistical difference (*p* <0.05) in body weight between WTDSS and Gal3KODSS mice. Hence, animals with complete deletion of *Gal3* showed a 12% increase in body weight (*p* <0.05, compared with WTDSS mice; see Supplementary Appendix S1 for multifactor ANOVA analysis). DAI score also revealed that the WTDSS mice were much more affected as compared with Gal3KODSS mice. Results ranged from 2.25 ± 0.5 in WTDSS to 0.5 ± 0.57 in Gal3KODSS (*p* <0.001; Figure 1B; see Supplementary Appendix S1 for multifactor ANOVA analysis), indicating that the Gal3KODSS mice scarcely had clinical symptoms. Regarding the colon length shortening and histological scoring, Figure 1C reveals that the colon length significantly decreased in both WTDSS and Gal3KODSS mice compared with their respective controls without DSS treatment (*p* <0.001). However, colon shortening was significantly higher in WTDSS mice as compared with Gal3KODSS mice (*p* <0.001; see Supplementary Appendix S1 for multifactor ANOVA analysis.). Histological analysis showed colon inflammation in both WTDSS and Gal3KODSS mice, but in WT was much more significant ranging from 12.1 ± 1.4 in WTDSS to 6.2 ± 2.3 in Gal3KODSS (*p* <0.001; Figures 1D,E; see Supplementary Appendix S1 for multifactor ANOVA analysis). Highlight that in Gal3KODSS mice loss of crypts and infiltration of immune cells was more severe in the colon wall than that seen in WTDSS (Figure 1D). RT-qPCR experiments showed an increase in both cytokines with the DSS treatment in both types of mice, but in the Gal3KO, compared to WT, the levels of IL-1β were significantly lower, ranging from 783.9 ± 9.2 in WTDSS to 314.2 ± 82.9 % in Gal3KODSS (*p* <0.001; Figures 1F,G; see Supplementary Appendix S1 for multifactor ANOVA analysis). According to all these results, Gal3KODSS mice developed a much milder acute colitis compared to WTDSS mice. Thus, *Gal3* deletion might protect against colitis development and associated peripheral inflammation.

### Effect of the Absence of *Gal3* on Cell Proliferation and Goblet Cells in the Colon of Dextran Sulphate Sodium -Treated Mice

We then tested whether the decreased susceptibility of the Gal3KODSS mice was associated with processes involved in maintaining the intestinal barrier that prevent the colitis, such as epithelial renewal and mucus production. For that, we evaluated cell proliferation rate and the presence of mature goblet cells in the distal colon epithelium of WT and Gal3KO mice with and without DSS treatment.

**FIGURE 2:**
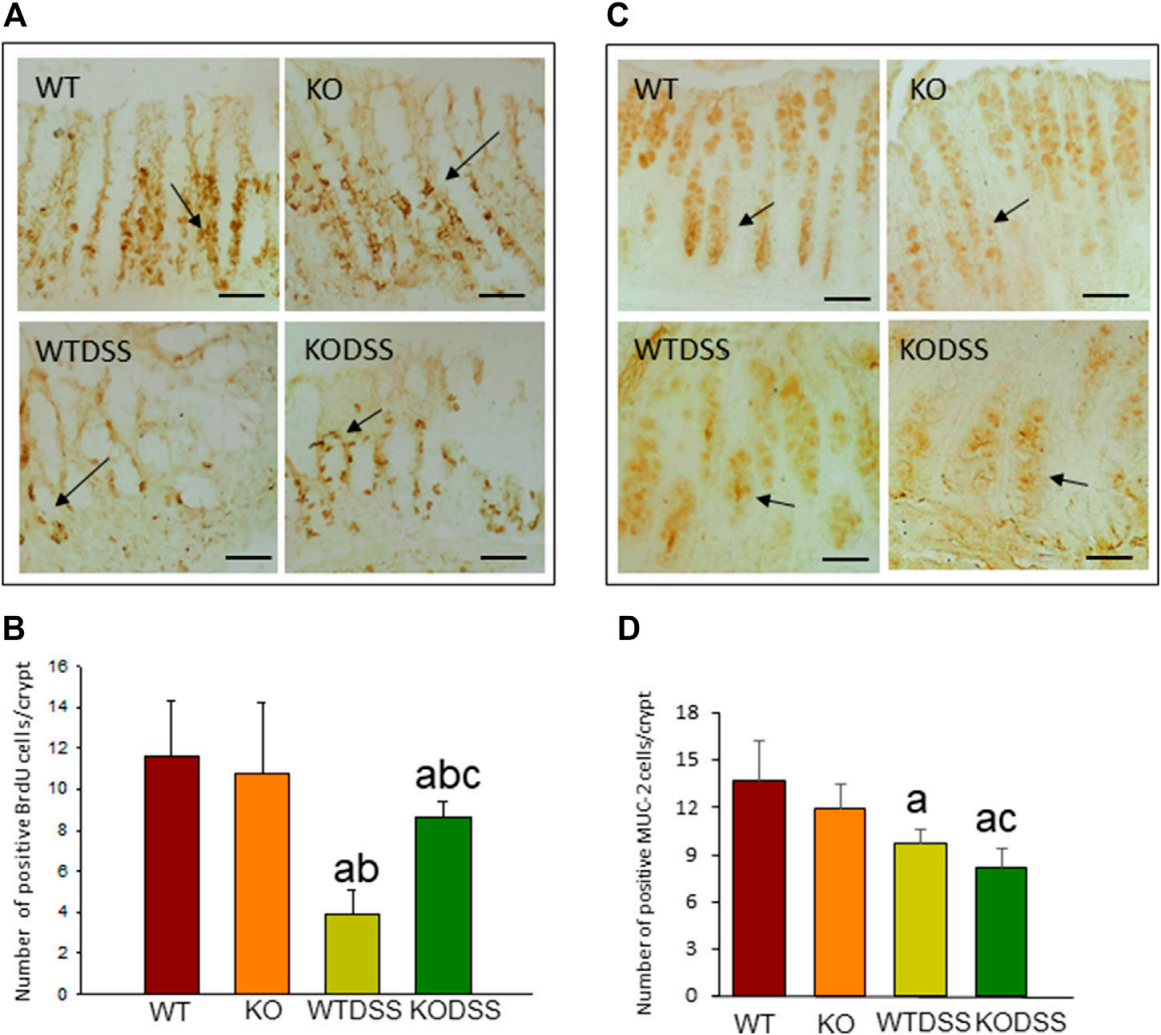
Effect of the DSS treatment on cell proliferation rate and goblet cells in the colon of WT and Gal3KO mice. Representative distal colon sections of cell proliferation rate assessment **(A)**. Examples of BrdU-labelled cells in the crypts are indicated by arrows. Scale bar = 20 µm. Cell proliferation rate expressed as number of BrdU positive cells per colonic crypt **(B)**; Representative distal colon sections of stained goblet cells **(C)**. Arrows indicate MUC-2-labelled goblet cells in the crypts. Quantification is expressed as the number of MUC-2 positive goblet cells per colonic crypt **(D)**. Scale bar: 100 µm. Results are mean ± SD of 10 mice per experimental group. Statistical analysis: One-way ANOVA followed by the Fisher’s LSD *post hoc* test for multiple comparisons was used, with *α* = 0.05: **(a)**, compared with the WT group; **(b)**, compared with the KO group; **(c)**, compared with the WTDSS group; *p* < 0.001 for BrdU, *p* < 0.05 for MUC-2. Abbreviations: WT, wild type mice; WTDSS, wild type mice treated with DSS; KO, Gal3 knockout mice; KODSS, Gal3 knockout mice treated with DSS.

BrdU incorporation into DNA was measured to evaluate epithelial cell proliferation rate, as described in Methods. The results showed that BrdU is mainly observed in the nuclei of the cells located in the lower part of the crypts where proliferation occurs (Figure 2A). In the Figure 2B, the quantification revealed that DSS-induced colitis decreased cell proliferation rate in both, WT and Gal3KO, being this diminution greater in WTDSS mice and therefore indicating that *Gal3* deletion significantly lessened the effect caused by DSS treatment (from 3.85 ± 1.21 in WT to 8.60 ± 0.77 in Gal3KO; *p* < 0.001; see Supplementary Appendix S1 for multifactor ANOVA analysis).

We next evaluated mature goblet cells by immunohistochemical detection of MUC-2 as a marker (Figure 2C). The quantification of number of MUC-2 positive cells per crypt (Figure 2D) revealed that the mature goblet cells and therefore MUC-2 production in the colonic mucosa diminished in the DSS-treated WT and Gal3KO mice (*p* <0.01), but it decreased significantly more in Gal3KO (from 9.7 ± 0.9 in WT to 8.2 ± 1.2 in Gal3KO; *p* <0.05; see Supplementary Appendix S1 for multifactor ANOVA analysis) indicating that *Gal3* deletion, in this case, increased the effect of DSS treatment.

### Peripheral Inflammation Induced by Lipopolysaccharide Injection Causes Microglial Activation in the Ventral Mesencephalon

**FIGURE 3:**
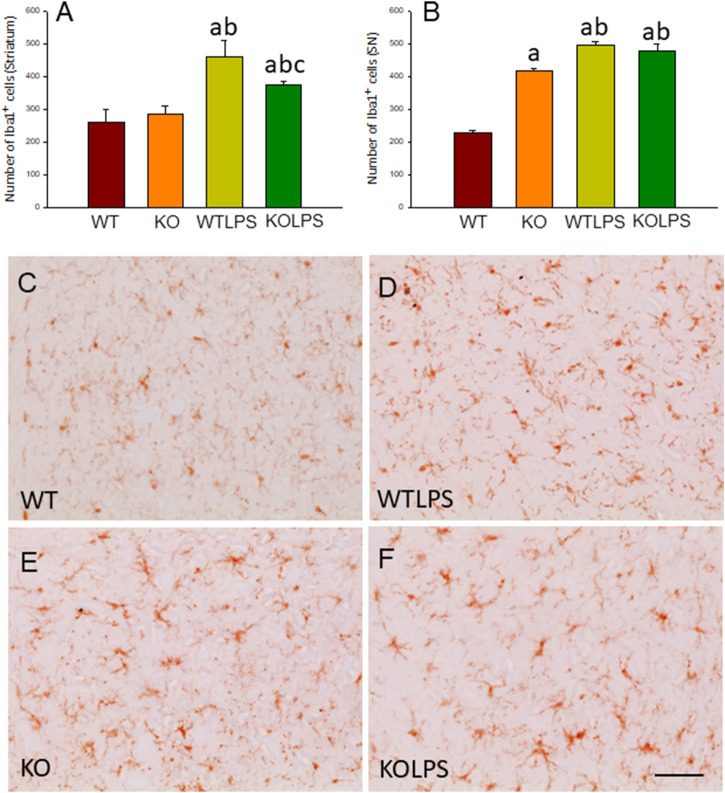
Evaluation of the microglial activation induced by LPS treatment in WT and Gal3KO mice. The effect of LPS was evaluated in striatum **(A)** and SN **(B)** from WT and Gal3KO mice. Results are mean ± SD of at least three mice per experimental group, expressed as the number of Iba1^+^cells per mm^2^. Statistical analysis: One-way ANOVA followed by the Fisher’s LSD *post hoc* test for multiple comparisons was used, with *α* = 0.05: **(a)**, compared with WT group; **(b)**, compared with KO group; **(c)**, compared with the WTLPS group; *p* < 0.001 **(C–F)** Representative immunostaining from striatum sections of the different experimental groups. Scale bar: 20 μm. Abbreviations: WT, wild type mice; WTLPS, wild type mice treated with LPS; KO, Gal3 knockout mice; KOLPS, Gal3 knockout mice treated with LPS.

**FIGURE 4:**
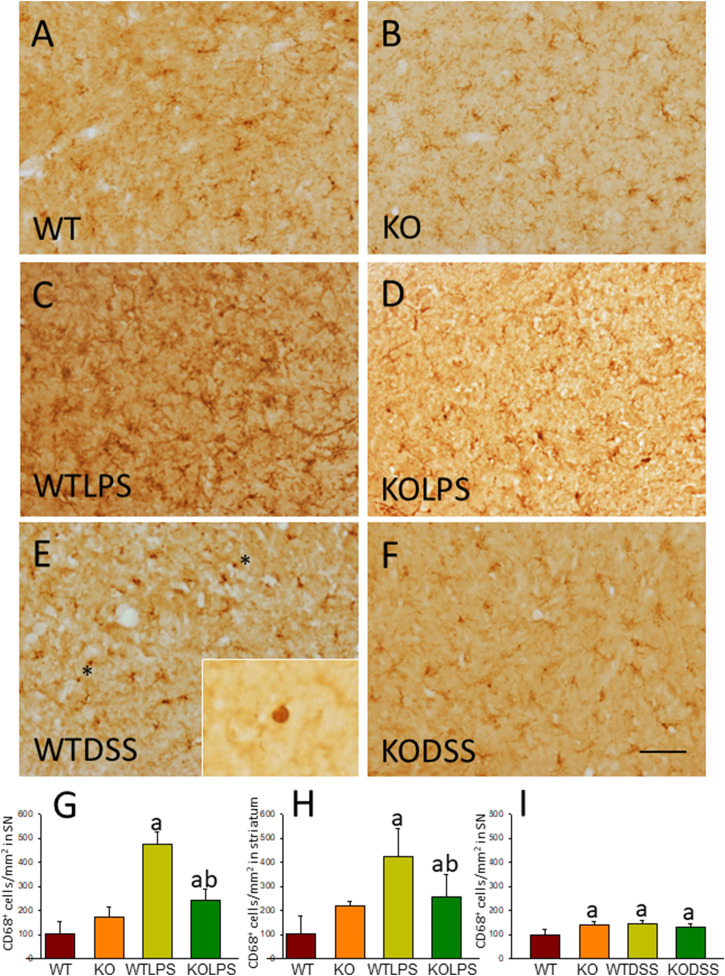
CD68 expression in LPS and DSS treated mice. Representative immunostaining from sections of the different treatments assayed. SN of WT **(A)** animals show a normal pattern of CD68 expression. Gal3KO **(B)** animals show a slight increase in the expression of CD68 in SN. WTLPS animals **(C)** show an increase in the number of CD68 positive cells while the absence of Gal3 in Gal3KOLPS **(D)** animals reduces this induction. Treatment with DSS in WT animals **(E)** also increases the number of CD68 positive cells. Notice the amoeboid morphology typical of highly reactive microglia in WT animals treated with DSS. Absence of Gal3 in DSS treated animals **(F)** do not reduce the number of CD68 positive cells but clearly avoid the presence of highly activated microglia with amoeboid morphology. Scale bar: 125 μm. **(G)** Quantification of the density of CD68 positive cells in SN of LPS treated mice. **(H)** Quantification of the density of CD68 positive cells in the striatum of LPS treated mice. **(I)** Quantification of the density of CD68 positive cells in SN of DSS treated mice. Results are mean ± SD of at least three animals per experimental group, expressed as the number of CD68 positive cells per mm^2^. Statistical significance: One-way ANOVA followed by the Fisher’s LSD post hoc test for multiple comparisons was used, with *α* = 0.05: **(a)**, compared with the WT group; **(b)**, compared with the WTLPS group; *p* < 0.05. Abbreviations: WT, wild type mice; WTLPS, WT animals treated with LPS; KOLPS, Gal3 knockout mice treated with LPS; WTDSS, wild type mice treated with DSS; KO, Gal3 knockout mice; KODSS, Gal3 knockout mice treated with DSS.

**FIGURE 5:**
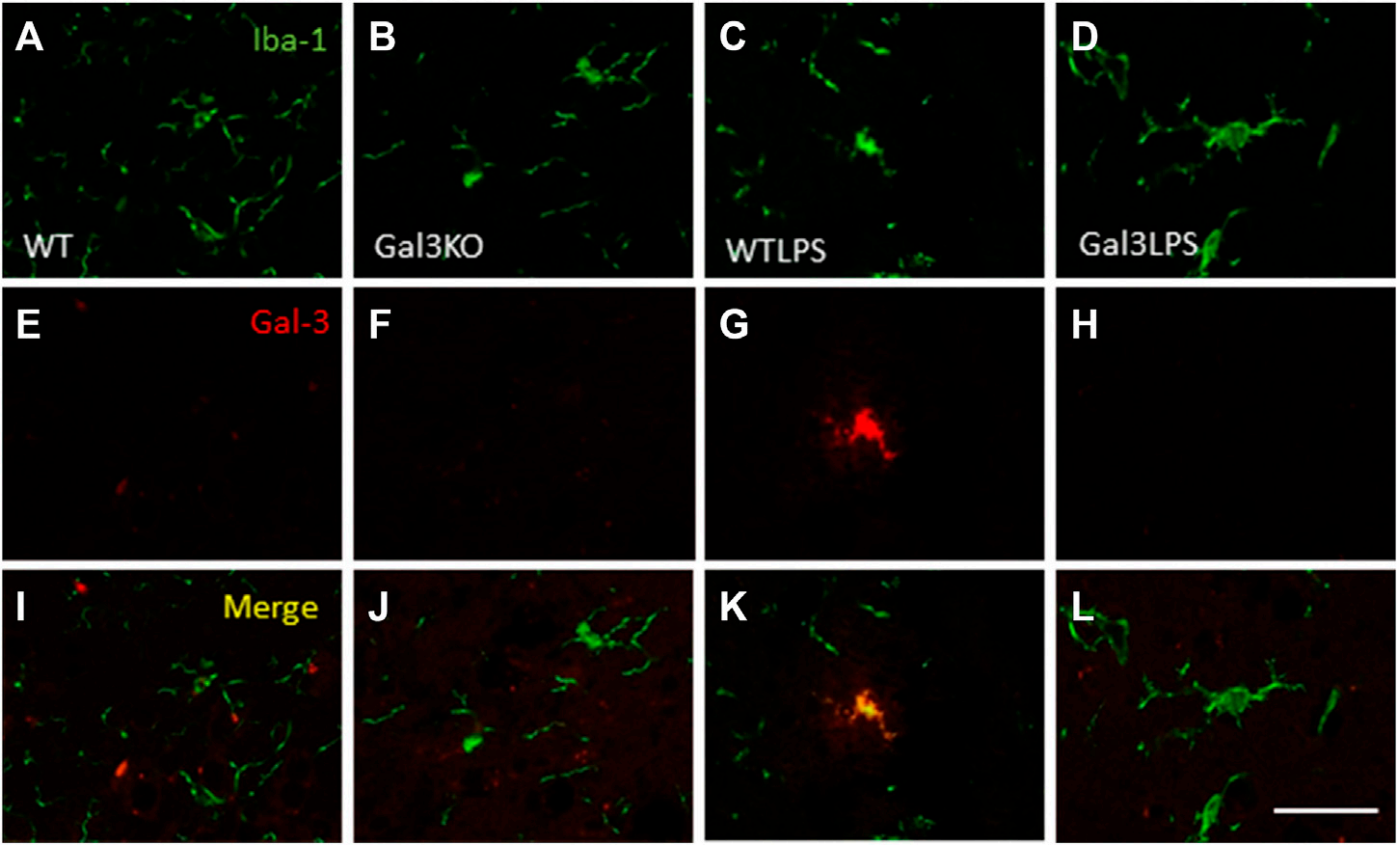
Expression of Gal3 in LPS-activated microglia. Representative images from sections of the different treatments assayed showing immunostaining of Iba1 (green) and Gal3 (red). Only a few Iba1 positive cells co-localized with Gal3 (yellow) in animales WT treated with LPS (K), indicating a not possible DAM phenotype. Scale bar: 75 μm. Abbreviations: WT, wild type mice; WTLPS, wild type mice treated with LPS; Gal3KO, Gal3 knockout mice; Gal3KOLPS, Gal3 knockout mice treated with LPS.

To study the role of peripheral inflammation on the brain, we first took advantage of an established model of peripheral inflammation based on four consecutive daily i.p. doses of LPS and then studied the microglial activation at both cellular and molecular levels. Using immunohistochemistry against Iba-1 (a pan microglia marker) we found that i.p. injection of LPS increased the number of microglial cells 5 days after the beginning of the treatment, in both the striatum (with an increase of 77.8% with respect to the control group, *p* <0.001; Figures 3A,C,D) and SN (with an increase of 118% with respect to the control group, *p* <0.0001; Figure 3B; see Supplementary Appendix S2 for multifactor ANOVA analysis) from WT animals. Although this increase in microglial cells in response to systemic LPS injection has been previously shown in striatum and cortex (Beier et al., 2017); (García Dominguez et al., 2018), we have extended these results by studying the SN, the most affected structure in PD. The study of microglial activation using immunohistochemistry against CD68 showed that LPS induces an increase of four fold in the number of CD68 positive cells, both in SN (Figures 4C,D,G) and striatum (Figure 4H). Co-localization of Iba-1 with Gal3 showed that only a few Iba1 positive cells co-localized with Gal3 in response to systemic LPS injections (Figure 5).

**FIGURE 6:**
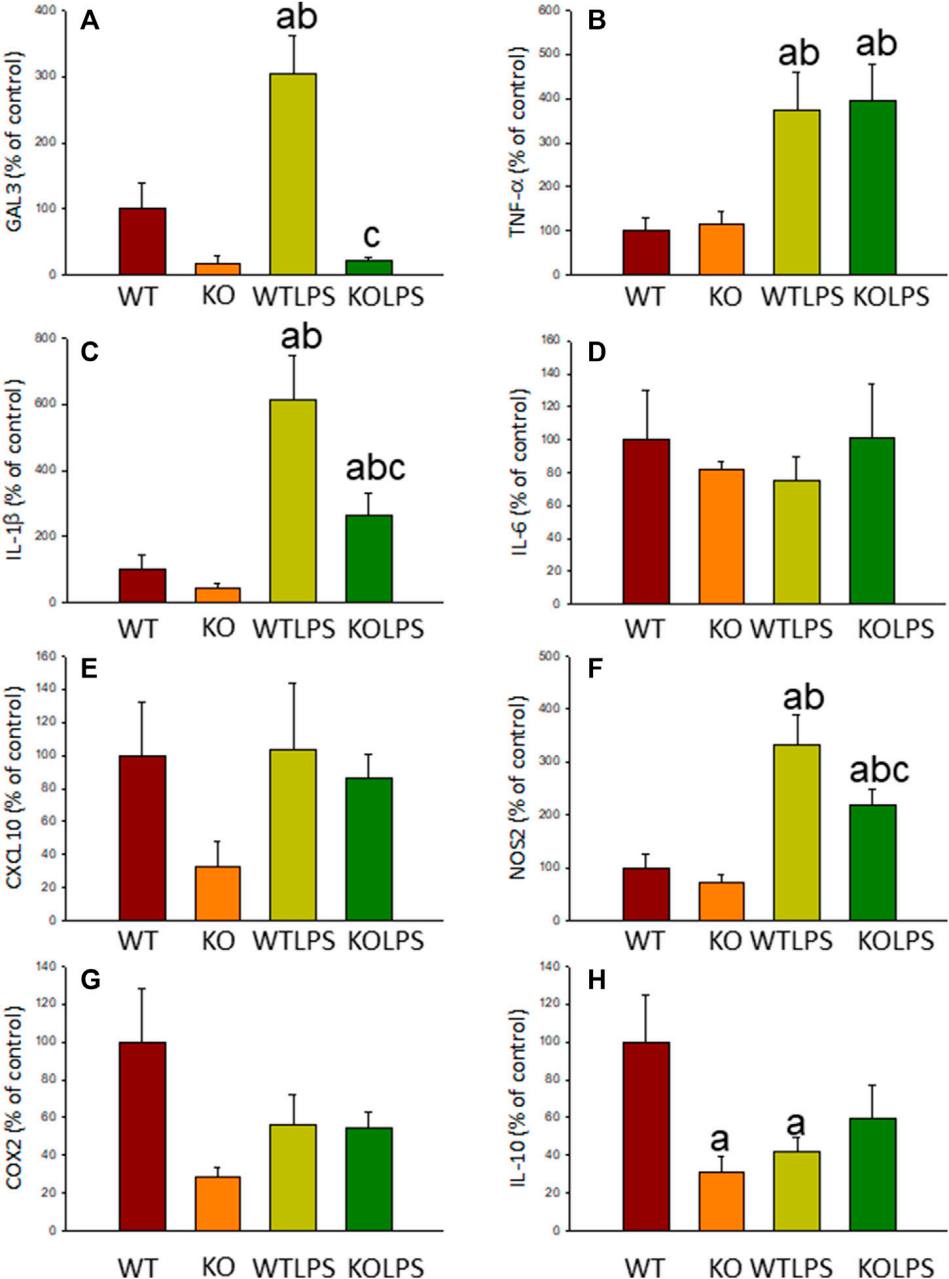
Effect of the LPS treatment on the expression levels of Gal3, TNF-α, IL-1β, IL-6, CXCL10, NOS2, COX2 and IL-10 in SN. Measurement of the effect of the treatment with LPS on the expression levels of mRNA of Gal3 **(A)**, pro-inflammatory mediators such as TNF-α **(B)**, IL-1β **(C)**, IL-6 **(D)**, CXCL10 **(E)**, NOS2 **(F)**, COX2 **(G)** and the anti-inflammatory mediator IL-10 **(H)** in the SN of mice from the different experimental groups by using RT-qPCR. Results are mean ± SD of at least three mice per experimental group, normalized to β-actin and expressed as percentage relative to the WT group. Statistical significance: One-way ANOVA followed by the Fisher’s LSD post hoc test for multiple comparisons was used, with *α* = 0.05: **(a)**, compared with the WT group; **(b)**, compared with the KO group; **(c)**, compared with the WTLPS group; *p* < 0.05. Abbreviations: WT, wild type mice; WTLPS, wild type mice treated with LPS; KO, Gal3 knockout mice; KOLPS, Gal3 knockout mice treated with LPS.

**FIGURE 7:**
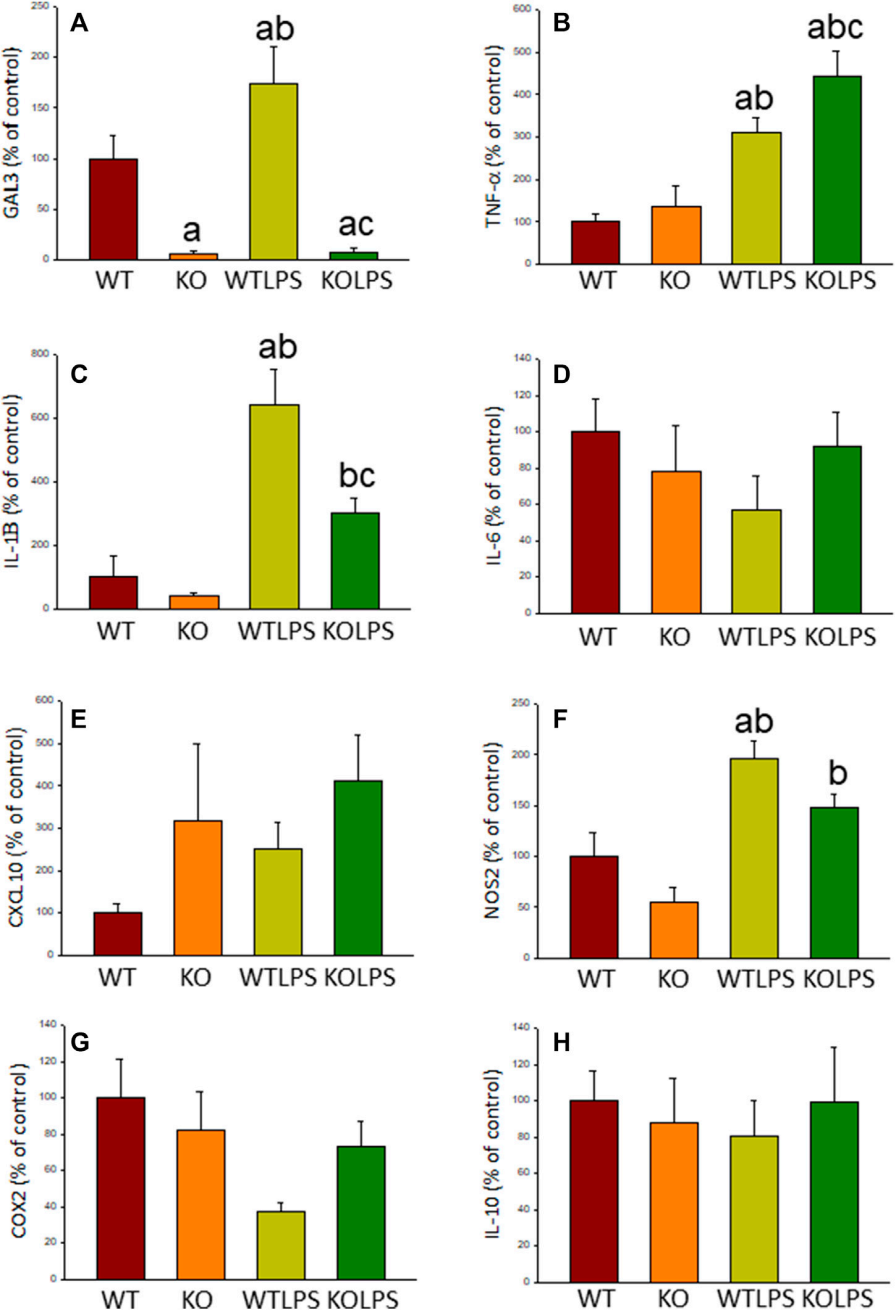
Effect of the LPS treatment on the expression levels of Gal3, TNF-α, IL-1β, IL-6, CXCL10, NOS2, COX2 and IL-10 in striatum. Measurement of the effect of the treatment with LPS on the expression levels of mRNA of Gal3 **(A)**, pro-inflammatory mediators such as TNF-α **(B)**, IL-1β **(C)**, IL-6 **(D)**, CXCL10 **(E)**, NOS2 **(F)**, COX2 **(G)** and the anti-inflammatory mediator IL-10 **(H)** in the striatum of mice from the different experimental groups by using RT-qPCR. Results are mean ± SD of at least three mice per experimental group, normalized to β-actin and expressed as percentage relative to the WT group. Statistical significance: One-way ANOVA followed by the Fisher’s LSD *post hoc* test for multiple comparisons was used, with *α* = 0.05: **(a)**, compared with the WT group; **(b)**, compared with the KO group; **(c)**, compared with the WTLPS group; *p* < 0.001. Abbreviations: WT, wild type mice; WTLPS, wild type mice treated with LPS; KO, Gal3 knockout mice; KOLPS, Gal3 knockout mice treated with LPS.

Microglial activation in the SN and striatum was also studied at the molecular level. Using RT-qPCR, we have evaluated the expression levels of Gal3 and some pro-inflammatory (TNF-α, IL-1β, IL-6, CXCL10, NOS2 and COX2) and anti-inflammatory (IL-10) mediators (Figures 6(SN),7 (striatum)). Most of these markers were chosen based on single-cell analysis of microglia revealing that upon systemic LPS challenge, microglia acquire a well-defined molecular signature characterized by strong up-regulation of different pro-inflammatory mediators including CXCL10, IL-6, IL-1β, COX2 and TNF (Krasemann et al., 2017). As expected, in SN only WT mice expressed Gal3 (Figure 6A). LPS-treatment increased the mRNA levels of Gal3 in the SN for up to three fold (Figure 6A; *p* <0.0001). Moreover, LPS injection produced a strong induction of mRNA expression levels of TNF-α, IL-1β and NOS2, ranging from 233.9 ± 54.8% for NOS2 to 610.9 ± 137.1% for IL-1β in WTLPS mice compared to WT (Figures 6B,C,F; *p* <0.01). In the case of the anti-inflammatory mediator IL-10, the treatment with LPS reduced significantly its expression level in WTLPS mice (*p* <0.05, compared with the control group; Figure 6H; see Supplementary Appendix S2 for multifactor ANOVA analysis). In striatum, LPS-treatment increased the mRNA levels of Gal3 (174 ± 36% with respect to the control group; Figure 7A; *p* <0.001). LPS injection also induced an upregulation of TNF-α, IL-1β and NOS2, ranging from 196.96 ± 16.72% for NOS2 to 641.69 ± 114.01% for IL-1β, in WTLPS mice compared to control group (Figures 7B,C,F; *p* <0.001; see Supplementary Appendix S2 for multifactor ANOVA analysis).

### Dextran Sulphate Sodium -Induced Acute Colitis Promotes Microglial Activation in the Ventral Mesencephalon

**FIGURE 8:**
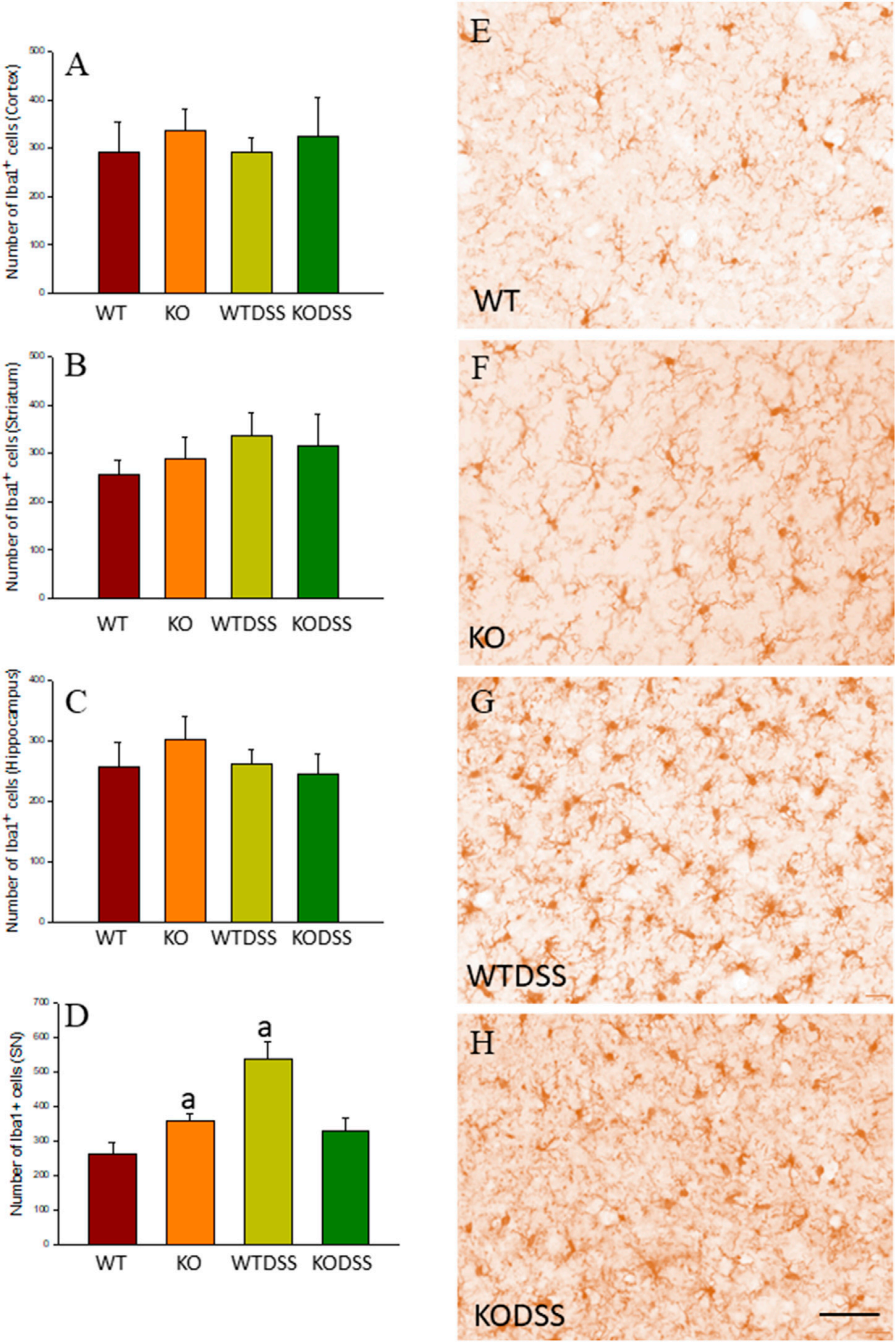
Evaluation of the microglial activation induced by DSS treatment in WT and Gal3KO mice. The effect of peripheral inflammation induced after 10 days of DSS treatment was evaluated in cortex **(A)**, striatum **(B)**, hippocampus **(C)** and SN **(D)** from WT and Gal3KO mice. Results are mean ± SD of at least three mice per experimental group, expressed as the number of Iba1^+^ cells per mm^2^. Statistical significance: One-way ANOVA followed by the Fisher’s LSD post hoc test for multiple comparisons was used, with *α* = 0.05: **(a)**, compared with the WT group; *p* < 0.001 **(E–H)** Representative immunostaining from SN sections of the different experimental groups. Scale bar: 20 μm. Abbreviations: WT, wild type mice; WTDSS, wild type mice treated with DSS; KO, Gal3 knockout mice; KODSS, Gal3 knockout mice treated with DSS.

**FIGURE 9:**
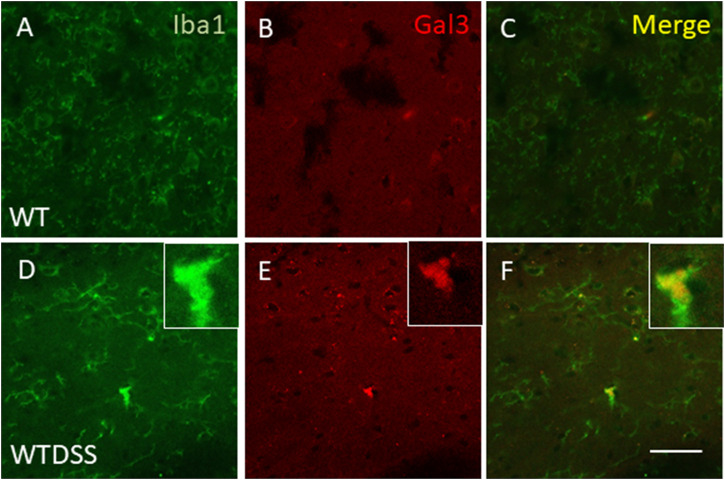
Expression of Gal3 in DSS-activated microglia. Representative images from sections of WT **(A–C)** and WTDSS **(D–F)** animals showing immunostaining of Iba1 (green) and Gal3 (red). As shown, only a few Iba1 positive cells co-localized with Gal3 (yellow) after the treatment with DSS **(F)**. Scale bar: 125 μm. Abbreviations: WT, wild type mice; WTDSS, wild type mice treated with DSS.

**FIGURE 10:**
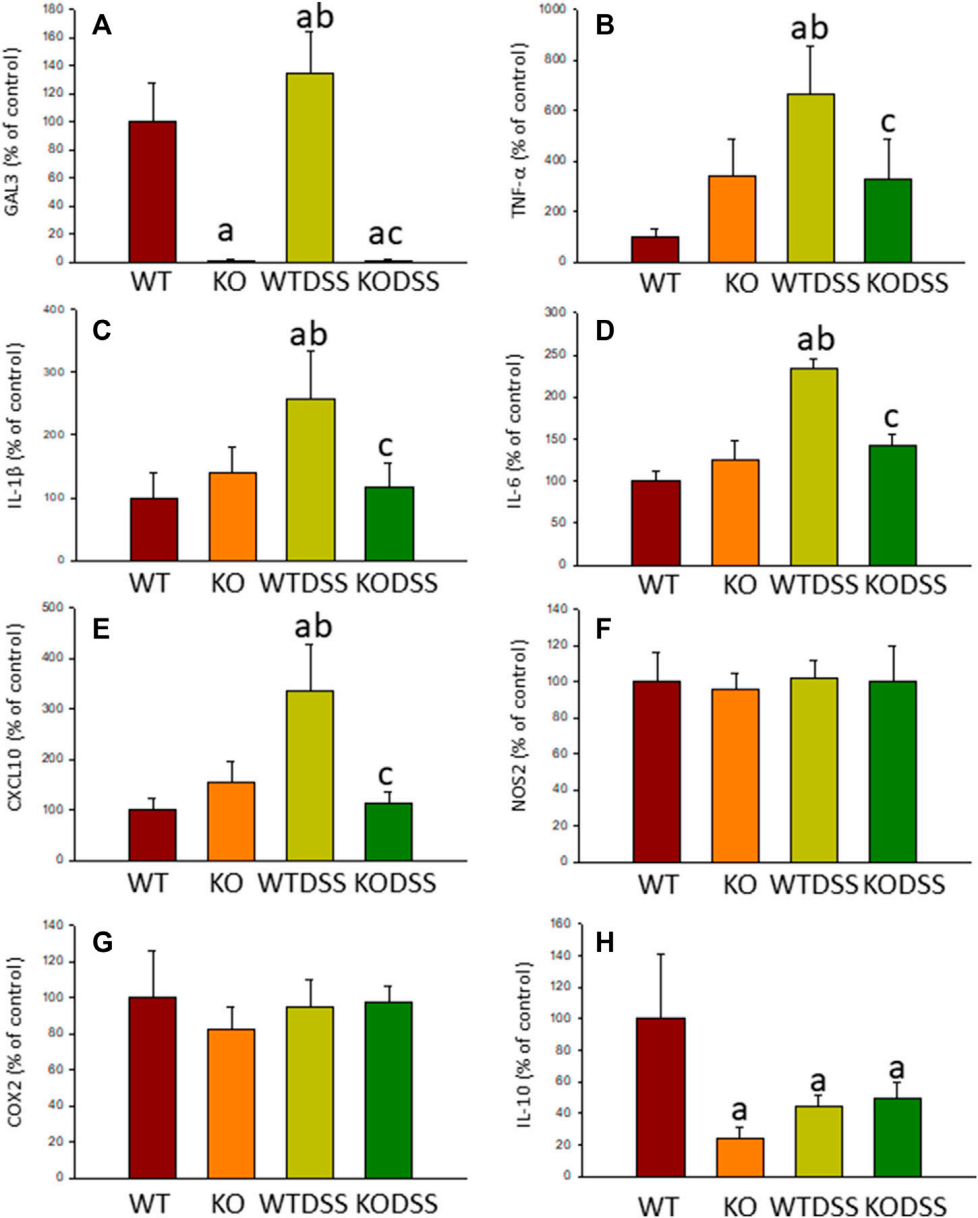
Effect of the DSS treatment on the expression levels of Gal3, TNF-α, IL-1β, IL-6, CXCL10, NOS2, COX2 and IL-10. Measurement of the effect of the treatment with DSS on the expression levels of mRNA of Gal3 **(A)**, pro-inflammatory mediators such as TNF-α **(B)**, IL-1β **(C)**, IL-6 **(D)**, CXCL10 **(E)**, NOS2 **(F)**, COX2 **(G)** and the anti-inflammatory mediator IL-10 **(H)** in the SN of mice from the different experimental groups by using RT-qPCR. Results are mean ± SD of at least three mice per experimental group, normalized to β-actin and expressed as percentage relative to the WT group. Statistical significance: One-way ANOVA followed by the Fisher’s LSD *post hoc* test for multiple comparisons was used, with *α* = 0.05: **(a)**, compared with the WT group; **(b)**, compared with the KO group; **(c)**, compared with the WTDSS group; *p* < 0.05. Abbreviations: WT, wild type mice; WTDSS, wild type mice treated with DSS; KO, Gal3 knockout mice; KODSS, Gal3 knockout mice treated with DSS.

As stated before, the effect of peripheral injection of LPS in the brain has been already studied (Beier et al., 2017). However, the effect of peripheral inflammation in the brain after DSS treatment has barely been explored. Therefore, we wanted to study neuroinflammation in different structures of CNS with implication in neurodegenerative diseases (cortex, hippocampus, striatum and SN), at both morphological and molecular levels. Using immunohistochemistry against Iba-1 (a pan microglia marker) and CD68 (a marker for microglial activation) we found that the SN was the only structure affected by our UC model (Figures 8A–D). Indeed, we failed to detect significant changes in microglia density in the striatum in response to acute DSS, contrary to systemic LPS challenge (Figure 8C). We have demonstrated that systemic inflammation induces a fast and transient microglia activation in striatum that may explain the different effects of acute colitis in striatum and SN (García Dominguez et al., 2018). Treatment with DSS induced a significant increase in the number of Iba-1 and CD68 cells in the SN of WT animals (203 and 146%, respectively, with respect to the control group; *p* <0.001; Figures 4E,I, 8D,E,G). When activated, microglial cells change their morphology from resting resident ramified microglia with two or three fine processes to round cells resembling tissue macrophages. After DSS treatment some CD68 positive cells showed a typical round morphology of chronically activated microglia (Figure 4E). However, co-localization of Iba-1 with Gal3 showed that only a few Iba1 positive cells co-localized with Gal3 (Figure 9). We failed to detect TREM2 expression by microglia after DSS treatment (data not shown). Since expression of TREM2 and Gal3 are features of disease-associated microglia (DAM) (Keren-Shaul et al., 2017); (Krasemann et al., 2017), we may conclude that nigral microglia get activated after acute gut inflammation but they acquire a phenotype different from classical DAM. As previously stated, different microglia modules have been identified under brain disease conditions (Friedman et al., 2018); (Krasemann et al., 2017). Expression of different pro-inflammatory markers potentially neurotoxic have been identified by single-cell analysis after acute systemic inflammation (Krasemann et al., 2017). Consequently, microglial activation in the SN was also studied at the molecular level to analyze if the pro-inflammatory module is induced by mesencephalic microglia under conditions of acute gut inflammation. Hence, using qRT-PCR, we evaluated the expression levels of Gal3 and some pro-inflammatory (TNF-α, IL-1β, IL-6, CXCL10, NOS2 and COX2) and anti-inflammatory (IL-10) mediators (Figure 10). As expected, only WT mice expressed Gal3 (Figure 10A; *p* <0.001). Interestingly, DSS-treatment increased the mRNA levels of Gal3 in the SN of WT animals. Moreover, the acute colitis produced a strong induction of mRNA expression levels of TNF-α, IL-1β, IL-6 and CXCL10, ranging from 233.5 ± 24.0% for IL6 to 663.5 ± 195.8% for TNF-α, in WTDSS mice compared to control group (Figures 10B–E; *p* <0.05). In the case of the anti-inflammatory mediator IL-10, the treatment with DSS reduced significantly its expression level in WTDSS mice (*p* <0.01, compared with the control group; Figure 10H). See Supplementary Appendix S1 for multifactor ANOVA analysis.

Absence of *Gal3* reduces microglial activation in the ventral mesencephalon from LPS or DSS treated mice.

We next studied the effect of deletion of *Gal3* on the neuroinflammation generated by LPS or DSS in each model. Immunohistochemistry against Iba-1 showed that the absence of Gal3 significantly decreased the neuroinflammation caused by LPS only in the striatum (1.2 fold with respect to the WTLPS, *p* <0.001; Figures 3A–D). However, it is interesting to notice that Gal3KO animals have more microglial cells than WT animals in SN. Therefore, if we compare Gal3KO with Gal3KOLPS animals, in SN, no statistical differences were found among these groups. However, multifactor ANOVA analysis showed that there is a positive interaction between genotype and treatment (see Supplementary Appendix S2). Consequently, the effect of LPS on the microglial population in the SN depends on the genotype, and the effect of genotype depends on the LPS/saline injection. Besides, the absence of Gal3 significantly decreased the number of microglial cells after DSS treatment in the SN (55.8125 ± 6.7 cells/mm^2^ respect to WTDSS; *p* <0.001; Figures 8D,G,H; see Supplementary Appendix S1 for multifactor ANOVA analysis). When we used CD68 to study microglial activation, we found that the number of cells expressing CD68 decreases around 50% in SN and striatum in Gal3KOLPS animals compared with the WTLPS group (Figures 4C,D,G,H). Although we did not find any statistical decrease in the number of CD68 positive cells in Gal3KODSS animals with respect to the WTDSS group (Figures 8E,F,I), microglial cells in Gal3KODSS mice did not show the typical round morphology of chronically activated microglia as seen in WTDSS animals (Figures 8E,F). Besides, we could not find any statistical differences between Gal3KO and Gal3KODSS animals. In this case, multifactor ANOVA analysis shows that there is an interaction between the two factors studied (genotype and DSS/water treatment).

Microglial activation in the SN and striatum was also studied at the molecular level, using RT-qPCR (Figures 5, 6, 9). Interestingly, the LPS and DSS-mediated induction of inflammatory mediators was prevented in GAL3KO animals. Hence, expression levels of IL-1β and NOS2 mRNA were decreased in the SN and striatum of Gal3KOLPS mice compared to WTLPS animals (more than 50% reduction for IL-1β and 25–35% for NOS2; Figures 5, 6). Even though Gal3 deletion failed to alter the number of Iba1 positive microglia in the SN in response to LPS as compared with WPLPS, the molecular analysis support the view that mesencephalic microglia acquire a pro-inflammatory phenotype, which is partially prevented in Gal3KOLPS. It is noteworthy that Gal3 deletion prevented the upregulation of all pro-inflammatory markers analyzed in the SN but not in the gut in response to DSS treatment. However, it should be considered the different nature of immune players (microglia vs peripheral immune cells) and focal vs distant areas of inflammation.

Moreover, expression levels of TNF-α, IL-1β, IL-6 and CXCL10 also decreased in the SN of Gal3KODSS mice compared with the WTDSS animals, with reductions ranging from 40% for IL-6–66% for CXCL10 (Figure 9). In the case of the anti-inflammatory mediator IL-10, the reduction induced by DSS treatment was also abolished in Gal3KO mice compared with its respective control (Figure 9H). See Supplementary Appendix S1, S2 for multifactor ANOVA analysis.

Peripheral inflammation induced by LPS injection and DSS treatment does not cause dopaminergic neuronal death in the ventral mesencephalon.

**FIGURE 11:**
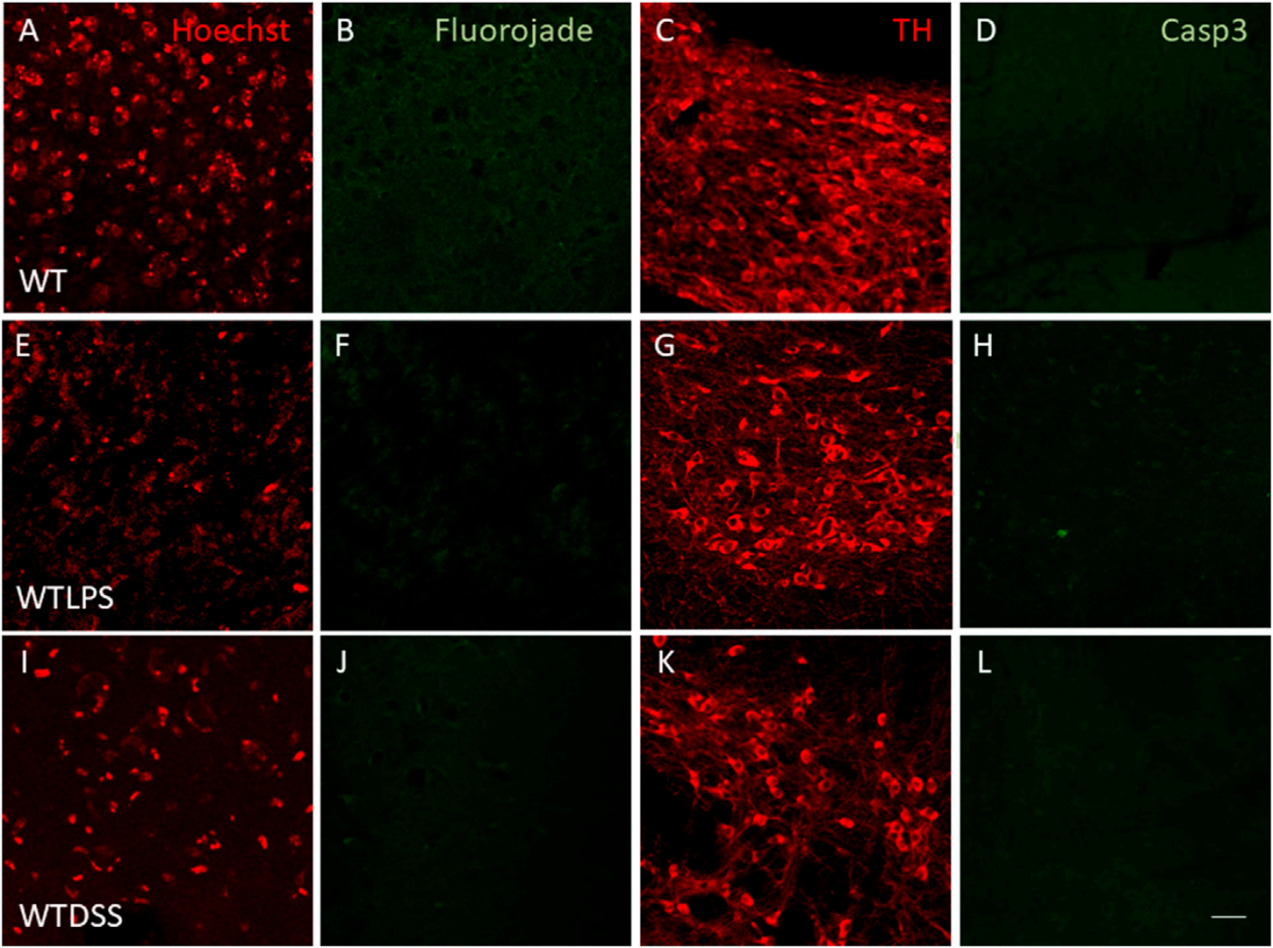
Effect of the treatment with LPS or DSS in the degeneration of dopaminergic neurons. Representative images from sections of WT, WTLPS and WTDSS animals. After 5 days of LPS treatment no signs of neurodegeneration or apoptosis were found **(E–H)** compared to the control group **(A–D)**. After 10 days of treatment with DSS neither neuronal degeneration **(J)** nor signs of apoptosis **(L)** were found compared to the control group **(A–D)**. Scale bar: 150 μm. Abbreviations: WT, wild type mice; WTLPS, WT animals treated with LPS; WTDSS, wild type mice treated with DSS.

We finally wanted to study if the microglial activation induced using the two models of peripheral inflammation was able to produce neuronal death in the SN, the main structure affected in PD. To this end we performed a Fluorojade C staining in order to assess whether LPS injection and/or DSS treatment induce neuronal degeneration. However, we did not find neurodegenerative events following LPS/DSS treatment (Figures 11E,F,I,J). We also performed a double immunofluorescence of TH (a dopaminergic neuronal marker) and caspase-3 (an apoptotic marker). Again, we did not find any sign of apoptosis in the TH positive neurons in none of the peripheral inflammation models used (Figures 11G,H,K,L).

## Discussion

The idea that peripheral inflammation could increase the damage in a previously inflamed brain is not new. We have previously demonstrated that the peripheral inflammation generated by an UC model, as well as a rheumatoid arthritis model, is able to increase the dopaminergic neuronal loss induced by intranigral injection of LPS (Villarán et al., 2010); (Hernández-Romero et al., 2012) or 1-methyl-4-phenyl-1,2,3,6-tetrahydropyridine (García-Domínguez et al., 2018), both models of PD. These studies strongly suggest that peripheral inflammation is a risk factor for the death of nigral dopaminergic neurons in response to different settings.

In the present work, we provide evidence that the inflammatory stimulus caused by either i.p. administration of LPS or by oral administration of DSS was able to produce by itself a similar pattern of neuroinflammation, especially in the SN. This is of special interest, since the SN is the main brain structure affected in PD, suggesting a potential role of peripheral inflammation in the development of this disease. The current results also demonstrate that Gal3 regulates the process involving inflammation either in the periphery or in the SN.

To prove our hypothesis, we first took advantage of an already established model of peripheral inflammation based on the i.p. injection of four consecutive doses of LPS (Beier et al., 2017). It has been previously demonstrated that this paradigm induces microglial activation in the striatum, along with a strong up-regulation of pro-inflammatory mediators in the first 5 days with no apparent neuronal loss, while at 19 days the microglial phenotype changes to an anti-inflammatory state coinciding with the beginning of the death of dopaminergic neurons (Beier et al., 2017). Therefore, we wanted to study the profile of microglial activation in the dopaminergic system, finding that, similarly to previous studies, the number of Iba1 positive cells increased in the striatum 5 days after the first injection of LPS. We completed this study measuring the number of microglial cells in the SN, and found that treatment with LPS increased the number of Iba1 and CD68 positive cells even more than in striatum.

We also studied the profile of cytokine expression in the SN 5 days after the first injection of LPS. Using qPCR techniques, we found an overexpression of genes typically related to the pro-inflammatory phenotype of microglia, including TNF-α, IL-1β and NOS2. Several authors have pointed out the key relevance of TNF-α in neurodegenerative conditions (Clark and Vissel 2021). Furthermore, (Qin et al., 2017), have shown that TNF-α levels remain elevated for up to 10 months after a single intraperitoneal dose of 5 mg/kg of LPS (Qin et al., 2007). In addition, various cytokines and immune cells may cause certain damage to dopaminergic neurons (Alrafiah et al., 2019); (Qin et al., 2016). Among them, IL-1 and IL-6 possess a potent pro-inflammatory effect. IL-1β and IL-6 gene polymorphisms play important roles in the occurrence and development of various diseases (Yousefi et al., 2018), including PD (Li et al., 2021).

We also wanted to know whether Gal3 is involved in this process. When we measured the expression of *Gal3* in the SN after the i.p. injections of LPS, we found a strong up-regulation of this protein, which suggests that Gal3 could be involved in the induction of a specific microglial phenotype after a peripheral inflammatory stimulus. Taking advantage of Gal3KO animals, we also found that absence of *Gal3* decreased the levels of some pro-inflammatory mediators in the SN and striatum, including IL-1β and NOS2, confirming the important role of Gal3 in driving this specific glial response.

To expand these results and go deeper in this issue, we also wondered if this pattern was shared by other pathological conditions that courses with peripheral inflammation. As stated before, the peripheral inflammation that accompanies IBD could be related to the development of PD and may be considered a risk factor for suffering this pathology (Brudek 2019). For this reason, we studied if the inflammatory environment induced by an acute colitis model in mice that resembles an active stage of UC could reach the brain and induce a similar profile of cytokines and if again, Gal3 is involved in this process. For this purpose, we first induced acute colitis through the oral administration of DSS in the drinking water, a model extensively used in the literature (Nascimento et al., 2020).

We first studied the clinical symptoms and colon state of DSS-treated mice and found that the treatment was able to produce an acute inflammatory process. This was supported by multiple markers including a decrease of body weight along with an increase in DAI score, colon shortening, damage in colon and increased mRNA levels of pro-inflammatory cytokines such as IL-1β and TNF-α. Interestingly, it has been previously shown that TNF-α is essential for the transfer of peripheral inflammation to the CNS (Qin et al., 2007).

In our experimental conditions, absence of *Gal3* led to lower inflammatory scores and levels of cytokines, preventing the appearance of symptoms of disease and attenuating the colonic inflammation induced by DSS, revealing an interesting possible role of Gal3 modulating peripheral inflammation. Results regarding Gal3 effects on colonic inflammation are contradictory. For instance, Simovic Markovic et al. (2016) showed an important pro-inflammatory role that Gal3 plays in events that accompany UC, while other authors found that Gal3 reduces the colon inflammation in animal models of experimental colitis (Tsai et al., 2016); (Lippert et al., 2015). More recently, it has been shown that the effects of *Gal3* deletion vary as colon inflammation progresses since Gal3 may direct the polarization of colonic macrophages towards inflammatory or anti-inflammatory phenotype (Volarevic et al., 2019). Moreover, Gal3 seems to be involved in processes that maintain the barrier in the colon. Similar to that reported by other authors (Araki et al., 2010), we observed decreased epithelial cell proliferation in the colon of DSS-treated mice. This alteration causes relevant leaks in the intestinal barrier because it prevents its restoration following injury, promoting colon inflammation and subsequent peripheral inflammation. In this condition, *Gal3* deletion attenuated the inhibition of the proliferation, suggesting that Gal3 could promote the inflammation by reducing epithelial renewal. Gal3 was found to inhibit cell proliferation in cultured endothelial cells (Zhang et al., 2018) but in other tissues and pathologies Gal3 promoted it (Viguier et al., 2014). On the other hand, according to our results, down-regulation of Gal3 decreases the intestinal epithelial intercellular adhesion (Jiang et al., 2014) which facilitates the proliferation.

Another interesting finding is related to the goblet cells. As expected, DSS treatment decreased the number of goblet cells (MUC-2 positive) in the colon (Kim and Ho 2010). In DSS-treated Gal3KO mice, this decrease was greater, indicating that Gal3 would facilitate MUC-2 production that forms the protective mucus layer contributing to prevent colon inflammation (Kim and Ho 2010). Our finding agrees with the up-regulation of MUC-2 transcription by Gal3 (Song et al., 2005). This effect would be opposite to the observations discussed so far and indicate again that Gal3 might play different roles associated with inflammatory processes as in other tissues and clinical contexts (Sciacchitano et al., 2018). These contradictory effects of Gal3 can be explained by the different subcellular localizations of Gal3 together with its possible post-translational modifications. Actually, Gal3 can be found in the cytoplasm, nucleus, and membranes (Shimura et al., 2004) and can be released into the extracellular space upon certain stimuli such as LPS (Li et al., 2008) and interferon γ (Jeon et al., 2010).

Once we examined the induction of acute colitis in our DSS model and the role that Gal3 plays in this process, we wanted to know whether this peripheral inflammation was able to reach the brain. For this purpose and using immunohistochemistry techniques, we looked for signs of neuroinflammation in several CNS structures typically involved in neurodegenerative diseases, such as cortex, hippocampus, striatum and SN. We found that peripheral inflammation induced a significant increase in the number of microglial cells only in the SN of DSS-treated animals. To shed more light on the nature of the neuroinflammation induced by the gut inflammation, we studied the transcriptional phenotype of microglial cells. Using qPCR, we found that DSS treatment induced the up-regulation of pro-inflammatory mediators in the SN, including again IL-1β and TNF-α. Nevertheless, the pro-inflammatory nature of these markers along with the increased number of CD68^+^ activated microglial cells supports the view that peripheral inflammation sustains pro-inflammatory microglia.

Once we studied the effect of peripheral inflammation caused by the colitis in the brain, we wanted to decipher the possible role that Gal3 could have in this process. Our results showed that genetic deletion of *Gal3* prevented the increase in microglial cells in the SN as well as the increase in the pro-inflammatory cytokines in response to DSS treatment. This effect could be mediated by the reduction of the gut inflammation, by a reduction of microglial activation, or more likely by the addition of the effects that the deletion of *Gal3* has in both peripheral and central inflammation.

An important question that emerges from our study is how Gal3 regulates brain inflammation associated with systemic inflammation. With the advent of single-cell RNA analysis of microglial cells in disease conditions, a TREM2-dependent microglia phenotype was identified and named either disease-associated phenotype (Keren-Shaul et al., 2017) or microglia neurodegenerative phenotype (Krasemann et al., 2017). A common feature to both phenotypes is upregulation of selective genes including *Trem2*, *Apoe*, *Clec7a*, *Itgax*, *Spp1* and *Lgals3* (Keren-Shaul et al., 2017); (Krasemann et al., 2017); (Mathys et al., 2017). There is controversy whether this microglial phenotype is protective or deleterious (Keren-Shaul et al., 2017); (Krasemann et al., 2017). It has been reasoned that given that loss-of-function mutations of TREM2 confer higher risk of suffering AD and PD, DAM is protective (Keren-Shaul et al., 2017). However, the picture is more complex than thought and, for instance, *Trem2* deletions have been shown to be protective and deleterious depending on the context (García-Revilla et al., 2019). Besides, we have demonstrated that Gal3, which is strongly upregulated in DAM (Krasemann et al., 2017); (Mathys et al., 2017), exerts a noxious role under different brain disease conditions including AD (Boza-Serrano et al., 2019), brain ischaemia (Burguillos et al., 2015) and traumatic brain injury (Yip et al., 2017). Whether the DAM phenotype is neuroprotective or deleterious, it is important to highlight that different microglial modules have been recently identified (Friedman et al., 2018). Among them, those associated with systemic inflammation have been characterized and shown to express high levels of classical pro-inflammatory genes including *Il6*, *Cxcl10*, *Il1b* and *Tnf* (Krasemann et al., 2017). Importantly, both acute models analyzed (LPS and DSS) clearly induced a pro-inflammatory module (Burguillos et al., 2015); (Krasemann et al., 2017). In addition, we failed to detect Gal3 and TREM2 immunoreactivity in activated microglia associated with both peripheral inflammatory conditions. Since DAM or MGnD phenotypes are characterized by strong upregulation of both markers (Keren-Shaul et al., 2017); (Krasemann et al., 2017); (Mathys et al., 2017), our data supports the view that under conditions of peripheral inflammation, Gal3 drives a microglia pro-inflammatory polarization through a peripherally acting mechanisms. Indeed, Gal3 is highly expressed in myeloid cells including monocytes, macrophages, dendritic cells and neutrophils (Díaz-Alvarez and Ortega 2017) and membrane-associated Gal3 plays immune-associated roles under acute and chronic inflammation (Díaz-Alvarez and Ortega 2017).

It is certainly intriguing why gut inflammation induces brain inflammation only in the SN. The maintenance of the gut barrier is synergistically coordinated by the immune system and by the gut microbiota (Donaldson and Mabbott, 2016); (Rooks and Garrett 2016). A breached intestinal barrier may allow gut microbiota–specific immune cells to act as systemic mediators able to penetrate the CNS and hence causing neuroinflammation (Buscarinu et al., 2017); (Pröbstel et al., 2020). Besides, the involvement of the vagus nerve in selectively driving immune response in SN should not be discarded. Indeed, the possibility that PD starts in the ENS and then spreads to the brain via the vagus nerve is consistent with Braak’s hypothesis (Braak et al., 2002); (Braak et al., 2003); (Braak et al., 2007).

In conclusion, we provide evidence that peripheral inflammation associated with systemic LPS and gut inflammation polarizes microglia towards a pro-inflammatory phenotype, potentially neurotoxic. Our results support the view that mice lacking Gal3 developed a much milder acute colitis in response to DSS treatment compared to WT mice. Hence, Gal3 deletion might protect from both colitis development and associated peripheral inflammation. Our data does not sustain a classical DAM phenotype under conditions of acute systemic inflammation (both models) and suggest a prominent role of Gal3 acting peripherally. It is certainly straightforward the selectivity of gut inflammation to solely drive inflammation in the ventral mesencephalon. Given the anatomical gut-brain connection through the vagus nerve, the involvement of this pathway should be considered in spreading inflammation from gut to brain. Indeed, epidemiological studies have demonstrated that IBD patients had an increased risk of suffering subsequent PD (Lin et al., 2016); (Peter et al., 2018); (Villumsen et al., 2019); (Weimers et al., 2019). Finally, we demonstrate that Gal3 is upregulated in the ventral mesencephalon under conditions of acute inflammation. We should consider that elevated serum levels of Gal3 from PD patients have been found (Yazar et al., 2019); (Cengiz et al., 2019) and Gal3 has been identified in the outer layers of Lewy Bodies from PD patients (Flavin et al., 2017), a clear indication that Gal3 may play a yet unidentified role if PD etiology and/or progression. Overall, Gal3 emerges as an important regulator of the immune response, both peripheral and central, and that inhibition of Gal3 may be a potential pharmacological approach to counteract diseases with an inflammatory base, such as PD.

## Data Availability

The raw data supporting the conclusions of this article will be made available by the authors, without undue reservation.

## Abbreviations

BrdU, 5-bromo deoxyuridine; DAI, disease Activity Index; DSS, dextran sulphate sodium; Gal 3, galectin-3; Gal3KO, gal3 null mutant mice; GWAS, genome-wide association studies; IBDs, inflammatory bowel diseases; IL, interleukin; i.p., intraperitoneally; LPS, lipopolysaccharide; MUC-2, anti-mucin-2; PBS, phosphate buffer saline; PD, Parkinson’s disease; RT, room temperature; RT-qPCR, real-time quantitative reverse transcription PCR; SN, substantia nigra; TBS, tris-buffered saline; TLR4, toll-like receptor; TNF, tumor necrosis factor; TREM2, triggering receptor expressed on myeloid cells 2; UC, ulcerative colitis; WT, wild type.
