# Supplementary material for: Galectin-3 Deletion Reduces LPS and Acute Colitis-Induced Pro-Inflammatory Microglial Activation in the Ventral Mesencephalon
Source: Front Pharmacol. 2021 Aug 18;12:706439. doi: 10.3389/fphar.2021.706439 (PMC8416309; doi:10.3389/fphar.2021.706439)
Supplement: Supplementary file 1 [file Table1.docx]

**APPENDIX 1**

Multifactor ANOVA Table for the variables and factors for DSS treatment

|  |  |  | Main effects |  | Interactions |
| --- | --- | --- | --- | --- | --- |
| Variables |  | A (WT/KO) | B (DSS/water) |  | AB |
| Iba-1 in Cortex | *F* ratio  Sig. level | 1.74 (1, 15)  0.2113 | 0.03 (1, 15)  0.8692 |  | 0.06 (1, 15)  0.8069 |
| Iba-1 in Striatum | *F* ratio  Sig. level | 0.05 (1, 15)  0.8336 | 4.76 (1, 15)  0.0497 |  | 1.15 (1, 15)  0.3042 |
| Iba-1 in Hippocampus | *F* ratio  Sig. level | 0.74 (1, 15)  0.4060 | 2.4 (1, 15)  0.1471 |  | 3.15 (1, 15)  0.1014 |
| Iba-1 in SN | *F* ratio  Sig. level | 6,90 (1, 13)  0.0253 | 32,61 (1, 13)  0.0002 |  | 51,57 (1, 13)  0.0000 |
| CD68 in SN | *F* ratio  Sig. level | 1.72 (1, 13)  0.2188 | 2.79 (1, 13)  0.1260 |  | 7.25 (1, 13)  0.0226 |
| COX2 in SN | *F* ratio  Sig. level | 0.92 (1, 17)  0.3544 | 0.39 (1, 17)  0.5411 |  | 1.71 (1, 17)  0.2114 |
| TNF in SN | *F* ratio  Sig. level | 0.55 (1, 16)  0.4705 | 14.45 (1, 16)  0.0022 |  | 15.76 (1, 16)  0.0016 |
| IL-1β in SN | *F* ratio  Sig. level | 3.79 (1, 17)  0.0720 | 0.17 (1, 17)  0.6875 |  | 4.95 (1, 17)  0.0430 |
| Gal3 in SN | *F* ratio  Sig. level | 56.34 (1, 17)  0.0000 | 1.20 (1, 17)  0.2913 |  | 1.18 (1, 17)  0.2948 |
|  |  |  |  |  |  |
| Arginase in SN | *F* ratio  Sig. level | 0.05 (1, 17)  0.8269 | 0.05 (1, 17)  0.8269 |  | 0.15 (1,17)  0.7047 |
|  |  |  |  |  |  |
| IL-6 in SN | *F* ratio  Sig. level | 4.53 (1, 15)  0.0548 | 23.69 (1,15)  0.0004 |  | 13.62 (1, 15)  0.0031 |
| CXCL10 in SN | *F* ratio  Sig. level | 2.54 (1, 15)  0.1367 | 3.43 (1, 15)  0.0886 |  | 7.01 (1, 15)  0.0213 |
| IL-10 in SN | *F* ratio  Sig. level | 10.54 (1, 15)  0.0070 | 1.92 (1, 15)  0.1916 |  | 13.86 (1, 15)  0.0029 |
| NOS2 in SN | *F* ratio  Sig. level | 0.00 (1, 15)  0.9961 | 0.37 (1, 15)  0.5569 |  | 0.85 (1, 15)  0.3745 |
| DAI | *F* ratio  Sig. level | 21 (1, 15)  0.0006 | 51.86 (1, 15)  0.0000 |  | 21 (1, 15)  0.0006 |
|  |  |  |  |  |  |
| Colon lenght | *F* ratio  Sig. level | 54.12 (1, 15)  0.0000 | 22.19 (1, 15)  0.0005 |  | 0.47 (1,15)  0.5045 |
|  |  |  |  |  |  |
| Weight loss | *F* ratio  Sig. level | 250.40 (1, 15)  0.0000 | 220.98 (1,15)  0.0000 |  | 233.13 (1, 15)  0.0000 |
| MUC2 | *F* ratio  Sig. level | 3.89 (1, 15)  0.0720 | 21.37 (1, 15)  0.0006 |  | 0.03 (1, 15)  0.8712 |
| BrdU | *F* ratio  Sig. level | 2.52 (1, 14)  0.1407 | 0.37 (1, 14)  0.5569 |  | 0.85 (1, 14)  0.3745 |
| Histological Score | *F* ratio  Sig. level | 27.78 (1, 19)  0.0001 | 246.7 (1, 19)  0.0000 |  | 27.78 (1, 19)  0.0001 |
|  |  |  |  |  |  |
| TNF in colon | *F* ratio  Sig. level | 0.07 (1, 22)  0.7916 | 8.92 (1, 22)  0.0076 |  | 0.08 (1,22)  0.7809 |
|  |  |  |  |  |  |
| IL-1β in colon | *F* ratio  Sig. level | 2.08 (1, 25)  0.1633 | 7.71 (1,25)  0.0110 |  | 2.20 (1, 25)  0.1518 |
