# Supplementary material for: Galectin-3 Deletion Reduces LPS and Acute Colitis-Induced Pro-Inflammatory Microglial Activation in the Ventral Mesencephalon
Source: Front Pharmacol. 2021 Aug 18;12:706439. doi: 10.3389/fphar.2021.706439 (PMC8416309; doi:10.3389/fphar.2021.706439)
Supplement: Supplementary file 2 [file Table2.docx]

**APPENDIX 2**

Multifactor ANOVA Table for the variables and factors for LPS treatment

|  |  |  | Main effects |  | Interactions |
| --- | --- | --- | --- | --- | --- |
| Variables |  | A | B |  | AB |
| Iba-1 in Cortex | *F* ratio  Sig. level | 6.62 (1, 14)  0.0259 | 1.19 (1, 14)  0.2988 |  | 1.78 (1, 14)  0.2089 |
| Iba-1 in Striatum | *F* ratio  Sig. level | 0.00 (1, 12)  0.9769 | 14.87 (1, 12)  0.0039 |  | 3.81 (1, 12)  0.0828 |
| Iba-1 in Hippocampus | *F* ratio  Sig. level | 17.07 (1, 13)  0.0020 | 10.92 (1, 13)  0.0079 |  | 2.27 (1, 13)  0.1628 |
| Iba-1 in SN | *F* ratio  Sig. level | 132.83 (1, 13)  0.0000 | 467.20 (1, 13)  0.000 |  | 194.08 (1, 13)  0.0000 |
| CD68 in SN | *F* ratio  Sig. level | 8.35 (1, 11)  0.0202 | 60.07 (1, 11)  0.0001 |  | 27.87 (1, 11)  0.0007 |
| CD68 in striatum | *F* ratio  Sig. level | 0.17 (1, 13)  0.6857 | 14.11 (1, 13)  0.0037 |  | 7.72 (1, 13)  0.0195 |
| COX2 in SN | *F* ratio  Sig. level | 4.75 (1, 19)  0.0446 | 0.31 (1, 19)  0.5865 |  | 4.23 (1, 19)  0.0563 |
| TNF in SN | *F* ratio  Sig. level | 0.09 (1, 19)  0.7740 | 19.88 (1, 19)  0.0004 |  | 0.00 (1, 19)  0.9493 |
| IL-1β in SN | *F* ratio  Sig. level | 7.31 (1, 17)  0.0171 | 24.01 (1, 17)  0.0002 |  | 3.76 (1, 17)  0.0731 |
| Gal3 in SN | *F* ratio  Sig. level | 26.92 (1, 19)  0.0001 | 8.83 (1, 19)  0.0090 |  | 7.94 (1, 19)  0.0124 |
|  |  |  |  |  |  |
| IL-6 in SN | *F* ratio  Sig. level | 0.03 (1, 15)  0.8737 | 0.02 (1,15)  0.9036 |  | 0.76 (1, 15)  0.4009 |
| CXCL10 in SN | *F* ratio  Sig. level | 2.36 (1, 19)  0.1438 | 1.10 (1, 19)  0.3095 |  | 0.83 (1, 19)  0.3747 |
| IL-10 in SN | *F* ratio  Sig. level | 2.56 (1, 19)  0.1290 | 0.83 (1, 19)  0.3761 |  | 7.20 (1, 19)  0.0163 |
| NOS2 in SN | *F* ratio  Sig. level | 4.28 (1, 19)  0.0552 | 30.42 (1, 19)  0.0000 |  | 1.67 (1, 19)  0.2152 |
| COX2 in striatum | *F* ratio  Sig. level | 0.30 (1, 19)  0.5916 | 4.57 (1, 19)  0.0483 |  | 2.60 (1, 19)  0.1264 |
|  |  |  |  |  |  |
| TNF in striatum | *F* ratio  Sig. level | 3.53 (1, 19)  0.0785 | 34.04 (1, 19)  0.0000 |  | 1.20 (1,19)  0.2893 |
|  |  |  |  |  |  |
| Gal3 in striatum | *F* ratio  Sig. level | 35.44 (1,9)  0.0000 | 3.03 (1,19)  0.1007 |  | 2.75 (1, 19)  0.1170 |
| IL-10 in striatum | *F* ratio  Sig. level | 0.03 (1, 19)  0.8732 | 0.03 (1, 19)  0.8611 |  | 0.45 (1, 19)  0.5097 |
| IL-6 in striatum | *F* ratio  Sig. level | 0.11 (1, 19)  0.7400 | 0.52 (1, 19)  0.4800 |  | 1.99 (1, 19)  0.1772 |
| CXCL10 in striatum | *F* ratio  Sig. level | 2.90 (1, 19)  0.1078 | 1.24 (1, 19)  0.2814 |  | 0.06 (1, 19)  0.8141 |
|  |  |  |  |  |  |
| NOS2 in striatum | *F* ratio  Sig. level | 7.05 (1, 19)  0.0173 | 29.60 (1, 19)  0.0001 |  | 0.01 (1, 19)  0.9239 |
|  |  |  |  |  |  |
| IL-1β in striatum | *F* ratio  Sig. level | 7.86 (1, 18)  0.0134 | 32.00 (1,18)  0.0000 |  | 3.78 (1, 18)  0.0708 |
